# Machine Learning-Assisted Evaluation of Topography-Dependent Cellular Responses in Meniscus-Mimetic Piezoelectric Nanofibrous Scaffolds

**DOI:** 10.3390/jfb17100532

**Published:** 2026-10-21

**Authors:** Jeong In Kim, Minyoung Kim, Soonchul Lee, Claire Haeyoon Lim, Wonchul Choi, Sung-Sahn Lee

**Affiliations:** 1 Department of Orthopedic Surgery, CHA Bundang Medical Center, CHA University School of Medicine, Seongnam-Si 13488, Republic of Korea; 2 Department of Anatomy, CHA University School of Medicine, Seongnam-Si 13488, Republic of Korea; 3 Yongsan International School of Seoul, Seoul 04347, Republic of Korea; 4 Department of Orthopedic Surgery, Incheon St. Mary’s Hospital, College of Medicine, The Catholic University of Korea, Incheon 21431, Republic of Korea

**Keywords:** meniscus tissue engineering, piezoelectric nanofibers, fiber topography, mesenchymal stem cells, machine learning

## Abstract

Electrospun nanofibrous scaffolds provide topographical cues that regulate cellular behavior; however, relationships between scaffold design and multidimensional biological responses remain difficult to define because structural and material parameters act simultaneously. Here, we fabricated meniscus-mimetic piezoelectric nanofibrous scaffolds with different fiber architectures and compositions and systematically evaluated their cellular responses using machine learning (ML). Poly(vinylidene fluoride) (PVDF)-based scaffolds with random, aligned, and radial fiber architectures were prepared, including composite scaffolds containing barium titanate (BaTiO_3_) and multi-walled carbon nanotubes (MWCNTs). Scaffold morphology and fiber organization were quantitatively characterized, and mesenchymal stem cells (MSCs) cultured on the scaffolds were analyzed by DAPI, F-actin, and SRY-box transcription factor 9 (SOX9) fluorescence imaging. Quantitative image analysis revealed scaffold-dependent differences in cellularity, nuclear morphology and orientation, cytoskeletal organization, and SOX9-associated phenotype, demonstrating distinct cellular responses to fiber topography and material composition. Scaffold and cellular parameters were subsequently integrated into a quantitative dataset to develop and compare multiple ML models for modeling scaffold-dependent cellular responses. The ML framework captured relationships between scaffold characteristics and multidimensional biological outcomes. These findings establish an experimental–computational approach for linking electrospun scaffold design with cellular behavior and provide a data-driven framework for the rational evaluation and optimization of meniscus-mimetic piezoelectric scaffolds.

## 1. Introduction

The meniscus is a fibrocartilaginous tissue with a highly organized extracellular matrix that plays essential roles in load distribution, shock absorption, joint stabilization, and lubrication of the knee. Its biomechanical function is closely related to the spatial organization of collagen fibers within the tissue [1,2]. Circumferentially oriented collagen bundles constitute the major structural framework and resist tensile hoop stresses generated during joint loading, whereas radial tie fibers traverse these bundles and contribute to resistance against longitudinal splitting [3,4]. In addition, variations in fiber orientation and organization across meniscal regions create distinct local microenvironments [5]. Reproducing these heterogeneous fibrous architectures is therefore an important consideration in the design of scaffolds intended to mimic the structural characteristics of the native meniscus [6,7].

Electrospinning is particularly suitable for constructing such fibrous microenvironments because it enables control over fiber morphology, orientation, and material composition at the micro- and nanoscale [8,9]. The morphological and structural characteristics of electrospun fibers are strongly influenced by solution properties and processing conditions. In particular, polymer concentration and solution viscosity affect molecular-chain entanglement and jet stability, whereas solution electrical conductivity influences charge transport, electrostatic stretching, and fiber formation. Processing parameters, including applied voltage, solution flow rate, tip-to-collector distance, temperature, and relative humidity, further regulate jet dynamics, solvent evaporation, and fiber solidification. These interconnected factors influence fiber diameter, morphological uniformity, interfiber pore size, and the overall porous architecture of electrospun scaffolds. The effects of solution chemistry, including acidity or alkalinity where applicable, may also be relevant depending on the solvent system and polymer formulation. By modifying electrospinning conditions and collector configurations, fibers can be generated with random, uniaxially aligned, or radially oriented architectures. These architectures provide distinct topographical cues that regulate cell–material interactions through contact guidance. Aligned fibers can induce cellular elongation and directional organization along the fiber axis, whereas random fibrous networks provide a comparatively isotropic environment. Radially oriented fibers introduce a spatially varying directional cue that can guide cellular organization along radial trajectories. Thus, controlling fiber orientation provides a means of generating distinct topographical environments representative of structural features found within the meniscal extracellular matrix. By modifying electrospinning conditions and collector configurations, fibers can be generated with random, uniaxially aligned, or radially oriented architectures [10]. These architectures provide distinct topographical cues that regulate cell–material interactions through contact guidance. Aligned fibers can induce cellular elongation and directional organization along the fiber axis, whereas random fibrous networks provide a comparatively isotropic environment. Radially oriented fibers introduce a spatially varying directional cue that can guide cellular organization along radial trajectories. Thus, controlling fiber orientation provides a means of generating distinct topographical environments representative of structural features found within the meniscal extracellular matrix [11].

In addition to fiber architecture, material properties can influence how cells respond to electrospun scaffolds [9,10,12]. Piezoelectric biomaterials are of particular interest for musculoskeletal tissue engineering because mechanical deformation can generate localized electrical signals within the cellular microenvironment [13,14,15]. Poly(vinylidene fluoride) (PVDF) is a widely investigated piezoelectric polymer owing to its electroactive crystalline phases, mechanical stability, chemical resistance, and compatibility with electrospinning [16,17,18]. Incorporation of functional nanomaterials such as barium titanate (BaTiO_3_) and multi-walled carbon nanotubes (MWCNTs) can further alter the structural and electromechanical characteristics of PVDF-based fibers. Accordingly, combining controlled fiber architectures with different PVDF-based compositions enables the generation of scaffold environments that differ in both topographical and material characteristics [10,19].

The increasing diversity of scaffold design parameters, however, makes systematic evaluation of scaffold–cell interactions increasingly complex [20,21,22]. Fiber orientation, morphology, processing conditions, polymer composition, and functional additives can simultaneously influence multiple aspects of cellular behavior [9,23,24]. Conventional scaffold evaluation generally examines individual biological outcomes, including cell attachment, proliferation, morphology, or expression of selected molecular markers. Although these measurements provide important information, no single parameter adequately represents the overall cellular response to a complex scaffold environment. Moreover, evaluating individual endpoints independently may obscure relationships among cellular morphology, spatial organization, and molecular responses that arise from differences in scaffold design [12,25,26].

Quantitative cellular imaging provides an opportunity to characterize these responses across multiple dimensions. Nuclear staining can be used to determine cell number while also providing quantitative information on nuclear morphology, elongation, and orientation [27,28]. F-actin organization reflects cytoskeletal adaptation to the underlying fibrous architecture and can therefore provide a direct measure of topography-dependent cellular organization. Molecular imaging can further complement these structural measurements [27,29]. In the context of fibrocartilaginous tissues, SOX9 is an important transcriptional regulator associated with chondrogenic activity and can serve as an additional molecular readout of the cellular response to the scaffold environment [30,31]. Rather than using SOX9 as a standalone indicator of meniscal differentiation, its expression can be considered together with cellularity, nuclear morphology, and cytoskeletal organization to construct a multidimensional description of scaffold-dependent cellular behavior. Integration of these imaging-derived parameters may therefore provide a more comprehensive representation of cell–scaffold interactions than conventional analyses based on individual endpoints [31,32].

Machine learning (ML) offers a useful approach for analyzing such multidimensional experimental datasets. ML algorithms can integrate multiple, potentially interdependent variables and identify relationships between scaffold characteristics and biological outcomes that may not be readily apparent from individual comparisons. Recent applications of ML in biomaterials research have included prediction of material properties, analysis of fabrication parameters, and modeling of cell–material interactions [33,34]. Electrospun scaffold systems are particularly suitable for this approach because differences in processing conditions, material composition, and fiber morphology can be quantitatively related to subsequent cellular responses [35,36]. Integrating experimental scaffold characterization with quantitative biological measurements may therefore enable a transition from conventional group-by-group comparisons toward a more systematic, data-driven analysis of scaffold–cell relationships.

Despite increasing interest in computational approaches for biomaterial design, the relationships among meniscus-mimetic fiber architecture, piezoelectric scaffold composition, and multidimensional cellular responses remain insufficiently understood. In particular, random, aligned, and radial fibrous architectures generate fundamentally different topographical environments, while variations in PVDF-based composition introduce additional material-dependent effects. Determining how these variables collectively relate to cellularity, morphology, cytoskeletal organization, and molecular responses requires an analytical approach capable of integrating multiple experimental parameters rather than relying on a single biological marker or outcome.

In this study, we fabricated a series of PVDF-based piezoelectric nanofibrous scaffolds with controlled random, aligned, and radial fiber architectures and different material compositions. Mesenchymal stem cells (MSCs) were cultured on the scaffolds, and scaffold-dependent cellular responses were quantitatively characterized using DAPI, F-actin, and SOX9 fluorescence imaging. Imaging-derived parameters representing cell number, nuclear morphology and orientation, cytoskeletal organization, and SOX9 expression were integrated to establish multidimensional cellular response profiles for the different scaffold environments. The resulting experimental dataset was subsequently incorporated into an ML framework to investigate relationships between scaffold characteristics and cellular outcomes and to assess the feasibility of computationally modeling scaffold-dependent responses. Through this combined experimental and data-driven approach, we sought to establish quantitative relationships between the design of meniscus-mimetic piezoelectric nanofibrous scaffolds and the cellular responses induced by their distinct topographical and material environments.

## 2. Experimental Section

### 2.1. Materials Preparation

Poly(vinylidene fluoride) (PVDF) pellets with an average molecular weight of approximately 530,000 and barium titanate (BaTiO_3_, BT) nanoparticles were purchased from Sigma-Aldrich (Seoul, Republic of Korea). Multi-walled carbon nanotubes (MWCNTs) were supplied by Nanosolutions, Inc. (Jeonju, Republic of Korea). Prior to use, the MWCNTs were chemically functionalized by treatment with nitric acid under reflux conditions, followed by sonication. The treated nanotubes were subsequently rinsed three times to remove residual acid and dried for 12 h. This treatment was performed to generate oxygen-containing functional groups, primarily hydroxyl and carboxyl groups, on the MWCNT surface. Acetone and N,N-dimethylformamide (DMF), obtained from Sigma-Aldrich (Republic of Korea), were used to prepare the electrospinning solutions. Components required to modify the rotating collector, including iron wires, cellophane tape, and a steering wheel-shaped conductive structure, were obtained from Cosmo-Tech (Jeonju, Republic of Korea). Electrospinning was performed using a 20-gauge needle and a 12-mL syringe purchased from NORM-JECT^®^ (Henke-Sass, Wolf GmbH, Tuttlingen, Germany).

### 2.2. Preparation and Electrospinning of PVDF-Based Fibrous Scaffolds

The PVDF concentrations used in this study were selected based on preliminary electrospinning optimization experiments conducted over a range of polymer concentrations starting from 13 wt%. At lower concentrations, particularly around 13 wt%, electrospinning produced extremely thin fibers and poorly consolidated deposits that could not be reliably recovered as self-supporting membranes or reproducibly organized into the intended fiber architectures. In contrast, PVDF concentrations of approximately 15–17 wt% resulted in relatively stable fiber formation with comparable fibrous morphologies. At 20 wt%, membrane fabrication remained feasible, although morphological irregularities, including flattened or partially fused fibers and film-like regions, became apparent. Accordingly, 16 wt% was selected as a representative condition for relatively uniform fiber formation, whereas 20 wt% was selected as a higher-concentration condition exhibiting distinct morphological characteristics while retaining membrane fabricability. These two concentrations were therefore used to examine the influence of polymer concentration on scaffold morphology and structural organization.

PVDF-based electrospinning solutions were subsequently prepared at the selected polymer concentrations (16 and 20 wt%) using a 1:1 (*w*/*w*) mixture of N,N-dimethylformamide (DMF) and acetone as the solvent system. At each polymer concentration, both pristine PVDF and PVDF/BaTiO_3_ (BTO)/multi-walled carbon nanotube (MWCNT) composite formulations were prepared. For the 16 wt% formulation, 10 g of PVDF pellets was dissolved in the mixed solvent to obtain the pristine PVDF solution. To prepare the corresponding composite formulation, 1.0 g of BTO nanoparticles and 0.7 g of carboxyl-functionalized MWCNTs (MWCNT-COOH) were incorporated into the PVDF solution. Similarly, the 20 wt% PVDF solution was prepared using 14 g of PVDF pellets, while the composite formulation was obtained by adding 1.4 g of BTO nanoparticles and 1.0 g of MWCNT-COOH. All prepared solutions were continuously stirred at 100 °C for 14 h and subsequently cooled to room temperature prior to electrospinning.

Fibrous scaffolds with random, uniaxially aligned, and radially oriented architectures were fabricated by modifying the electrospinning collector configuration. Random and aligned fibers were generated using a metallic collector covered with a polycarbonate film in combination with horizontally positioned copper wires and vertically arranged cellophane tape to regulate fiber deposition and orientation. For radial fiber organization, the collector was replaced with a ring-and-pin electrode configuration consisting of an iron ring and a metallic pin positioned at its center. This configuration generated a spatially distributed electric field that directed fiber deposition radially between the central pin and surrounding ring electrode.

Electrospinning was performed using a commercially available electrospinning system (ESR200 series, NanoNC Co., Ltd., Seoul, Republic of Korea) equipped with a high-voltage power supply, a programmable syringe pump, and an interchangeable collector assembly. The prepared solutions were loaded into syringes equipped with 20-gauge needles and supplied at a constant flow rate of 1 mL h^−1^. A DC voltage of 16 kV was applied, and the tip-to-collector distance was maintained at 150 mm. Electrospinning was conducted at 28 °C and approximately 70% relative humidity. Temperature and relative humidity were regulated using an air-conditioning and dehumidification system and continuously monitored with a thermohygrometer. After electrospinning, the fabricated PVDF and PVDF/BTO/MWCNT fibrous scaffolds were dried at 30 °C for 24 h before subsequent characterization and cell experiments.

### 2.3. Morphological Characterization

The surface morphology and fibrous architecture of the electrospun scaffolds were characterized using field-emission scanning electron microscopy (FE-SEM; JSM-7001F, JEOL Ltd., Akishima, Tokyo, Japan). Prior to imaging, the samples were sputter-coated with a 6-nm-thick platinum layer. SEM images were acquired at multiple randomly selected regions of each scaffold to evaluate fiber morphology, diameter, interfiber spacing, and fiber organization. Fiber diameter and interfiber distance were quantitatively measured from the SEM micrographs using ImageJ (version1.52p; National Institutes of Health, Dethesda, MD,USA), and the resulting measurements were expressed as mean ± standard deviation or presented as distributions, as appropriate.

Fiber orientation was further quantitatively evaluated from the SEM images using fast Fourier transform (FFT) analysis in ImageJ. The two-dimensional FFT patterns generated from the micrographs were used to assess the degree and directionality of fiber organization among the random, uniaxially aligned, and radially oriented scaffold architectures [37]. Random fibrous scaffolds exhibited broadly distributed orientation patterns, whereas aligned and radial scaffolds showed preferential directional organization corresponding to their designed fiber architectures. In addition, orientation-dependent false-color maps were generated from the SEM images to spatially visualize local fiber directions and facilitate comparison of fiber organization among the different scaffold configurations.

The incorporation and spatial distribution of BTO nanoparticles and MWCNTs within the PVDF composite fibers were further examined using transmission electron microscopy (TEM; JEM-2010, JEOL, Japan). For TEM analysis, PVDF/BTO/MWCNT fibers were directly collected onto carbon-film-coated 200-mesh nickel grids (Electron Microscopy Sciences, Hatfield, PA, USA) during electrospinning and subsequently examined at an accelerating voltage of 200 kV. TEM images were used to assess the morphology and distribution of the incorporated BTO nanoparticles and MWCNTs within the electrospun PVDF fibers.

### 2.4. Cell Culture and Seeding

Human bone marrow-derived mesenchymal stem cells (hBMSCs; PCS-500-012, ATCC, Manassas, VA, USA) were cultured in Dulbecco’s Modified Eagle Medium (DMEM) containing 20% fetal bovine serum (FBS) and 1% penicillin/streptomycin. The cells were maintained under standard culture conditions at 37 °C in a humidified atmosphere with 5% CO_2_, with the culture medium replaced every two days. For subsequent experiments, hBMSCs were seeded onto the prepared matrices at a density of 2 × 10^4^ cells per well in 1 mL of complete culture medium.

### 2.5. Cell Morphology Analysis

Cell morphology and organization on the different matrices were examined by fluorescence staining. Following culture, the samples were fixed with 4% paraformaldehyde and permeabilized with 0.3% Triton X-100. The actin cytoskeleton was stained using ActinGreen™ (Invitrogen, Waltham, MA, USA), and cell nuclei were counterstained with DAPI. Fluorescence images were acquired using a confocal laser scanning microscope (LSM 880, Carl Zeiss, Oberkochen, Germany). The resulting images were subsequently used to assess cell spreading, cytoskeletal organization, and topography-dependent cellular alignment.

### 2.6. Immunofluorescence Staining

Immunofluorescence staining was performed to assess the expression of SOX9, a chondrogenic marker, in hBMSCs cultured on the different matrices. After culture, the samples were fixed with 4% paraformaldehyde and permeabilized with 0.3% Triton X-100. Nonspecific binding was blocked with 1% bovine serum albumin (BSA) for 30 min, followed by overnight incubation with an anti-SOX9 primary antibody (1:200). After washing, the samples were incubated with an Alexa Fluor™ 594-conjugated anti-mouse IgG secondary antibody (1:1000), and the nuclei were counterstained with DAPI. Fluorescence images were obtained using a confocal laser scanning microscope (LSM 880, Carl Zeiss, Oberkochen, Germany). SOX9 expression was quantitatively assessed using ImageJ software (version 1.54) by measuring the mean fluorescence intensity (MFI) under identical image acquisition and analysis conditions across the experimental groups [38].

### 2.7. Computational Analysis and Machine Learning

Quantitative fluorescence imaging data were integrated with scaffold structural descriptors to systematically evaluate scaffold performance. DAPI fluorescence images were analyzed using Cellpose-based nuclear segmentation to quantify cell number and nuclear morphological features, including nuclear area, elongation, orientation, and alignment. F-actin fluorescence images were analyzed using pixel-based image analysis to quantify cytoskeletal coverage and cellular organization. A custom Python (version 3.11.16)-based image analysis pipeline was developed to automatically quantify scaffold fiber orientation from SEM images using fast Fourier transform (FFT) analysis and cellular alignment from F-actin images using structure-tensor-based analysis. The resulting orientation distributions were subsequently compared to quantify scaffold–cell alignment correspondence, providing a quantitative measure of the extent to which cellular organization followed the underlying scaffold fiber architecture.

Nuclear SOX9 expression was quantified within DAPI-defined nuclear masks following background correction, and the proportion of SOX9-positive nuclei was determined using a global Otsu threshold derived from the background-corrected nuclear SOX9 fluorescence intensity distribution. These quantitative cellular parameters were subsequently integrated into a Meniscus-Mimetic Scaffold Quality Index (MMS-QI), designed to provide an overall measure of scaffold-induced cellular performance. The MMS-QI comprised three major domains representing (i) cell quantity, (ii) cellular morphology and scaffold–cell alignment correspondence, and (iii) SOX9 expression. Individual parameters were normalized prior to integration, and the three domain scores were combined using a weighted arithmetic mean, with greater weight assigned to the SOX9 expression domain to emphasize the maintenance of a meniscus-associated chondrogenic phenotype.

Scaffold structural descriptors, including porosity, fiber diameter, pore size, and fiber orientation, were used as input variables to predict individual cellular responses and the integrated MMS-QI. Multiple linear and nonlinear regression algorithms were evaluated using a consistent cross-validation framework, and model performance was compared using standard regression metrics. Pearson correlation analysis was additionally performed to examine associations between scaffold structural characteristics and cellular response parameters. Given the limited number of scaffold formulations included in the present study, predictive modeling was considered exploratory and was primarily used to identify structure–response relationships and compare the relative predictive performance of different modeling approaches rather than to establish a broadly generalizable predictive model. Quantitative data are presented as mean ± standard deviation (SD), with the number of independent replicates specified in the corresponding figure or table captions.

## 3. Results and Discussion

### 3.1. Design and Fabrication of Meniscus-Mimetic Fibrous Architectures

The native meniscus possesses a hierarchically organized collagen network that is closely associated with its direction-dependent mechanical function [2]. The main body of the meniscus is dominated by circumferentially oriented collagen bundles, which resist tensile hoop stresses generated during joint loading, whereas radially oriented tie fibers traverse and interconnect the circumferential bundles and contribute to resistance against longitudinal splitting [4,5]. In addition, the superficial and lamellar regions exhibit fiber organizations distinct from those of the central circumferential network, resulting in a spatially heterogeneous fibrous architecture throughout the tissue [39,40]. Previous electrospinning-based meniscus studies have therefore incorporated combinations of random, circumferentially aligned, and radially oriented fibers to reproduce selected structural features of the native extracellular matrix [40].

**Figure 1:**
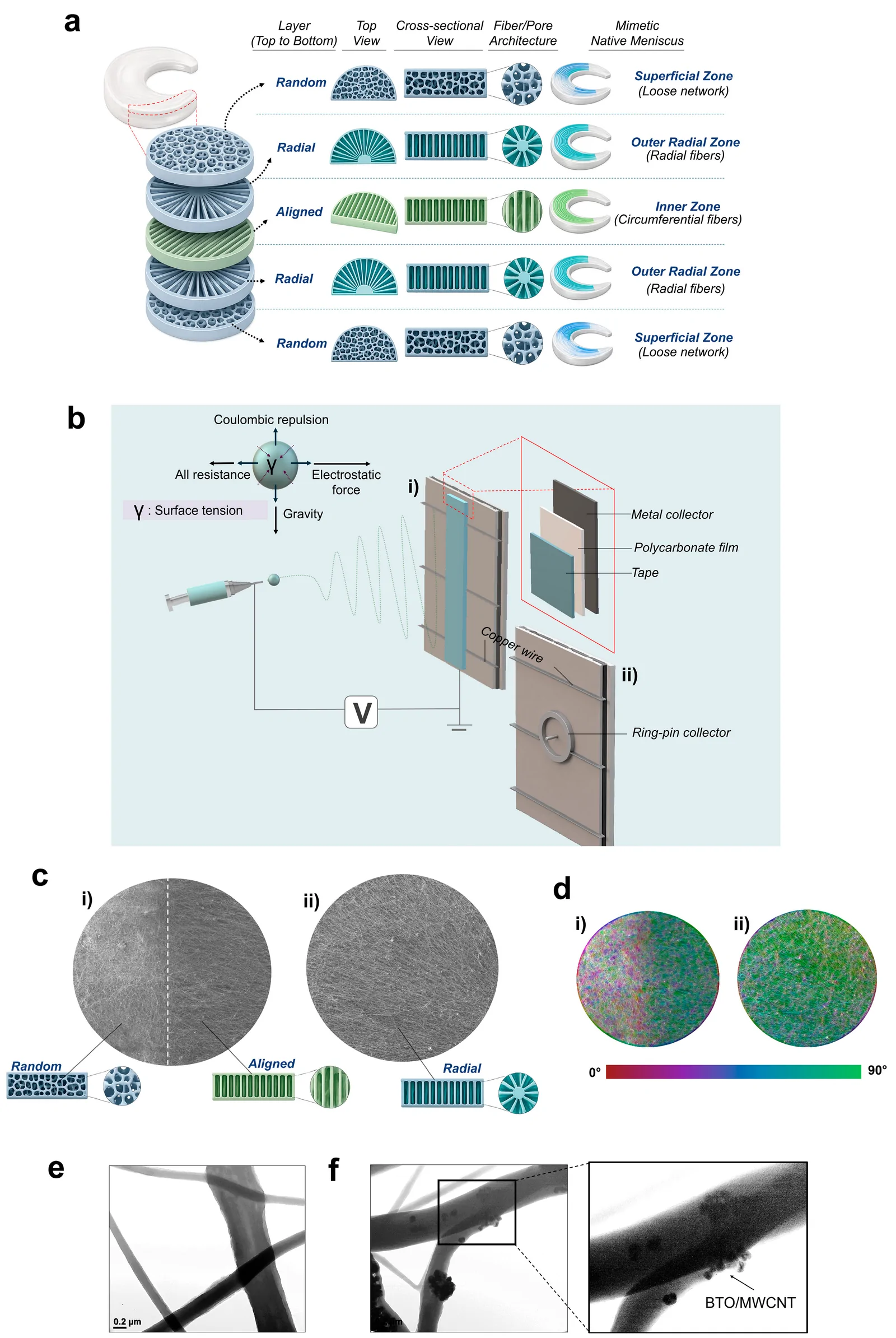
Design, fabrication, and morphological characterization of meniscus-mimetic PVDF-based nanofibrous scaffolds with controlled fiber architectures. (**a**) Schematic illustration of the meniscus-inspired hierarchical scaffold design. (**b**) Schematic illustration of the electrospinning process and collector configurations used to control fiber orientation. (**i**) Taping-assisted collector configuration used to generate spatially defined random and uniaxially aligned fibrous regions. (**ii**) Ring–pin collector consisting of a central pin and peripheral conductive ring used to generate radially oriented fibers through collector-mediated electric-field guidance. (**c**) Representative SEM images demonstrating collector-dependent fiber organization: (**i**) transition between random and aligned fibrous regions generated using the taping-assisted collector and (**ii**) radially oriented fibers generated using the ring–pin collector. (**d**) Orientation-dependent false-color maps generated from representative SEM images using OrientationJ in ImageJ, visualizing local differences in fiber directionality among random, aligned, and radial architectures; (**i**) transition between random and aligned fibrous regions generated using the taping-assisted collector; (**ii**) radially oriented fibers generated using the ring–pin collector. (**e**) Representative TEM image of a pristine PVDF electrospun fiber. (**f**) Representative TEM image of a PVDF/BTO/MWCNT composite fiber showing BTO nanoparticles and MWCNTs incorporated within the PVDF fiber matrix.

Based on this hierarchical organization, we established a simplified five-layer meniscus-mimetic architecture consisting of random–radial–aligned–radial–random fiber configurations as shown in Figure 1a. The random fibrous layers represented the comparatively multidirectional organization associated with the superficial fibrous environment, the central aligned layer reproduced the dominant circumferential fiber organization of the meniscal body, and the intervening radial layers represented radial tie-fiber-like architectures connecting the circumferential framework. The resulting structural concept was visualized from complementary top-view, cross-sectional, and fiber/pore architectural perspectives, and subsequently reconstructed into a crescent-shaped schematic to illustrate the spatial correspondence between the engineered fiber architectures and the native meniscus-inspired configuration (Figure 1a).

To experimentally generate these distinct topographical architectures, the electrospinning collector was modified to spatially control fiber deposition as presented in Figure 1b. In conventional electrospinning, application of a high electric potential induces charge accumulation at the polymer solution surface. As electrostatic stress overcomes surface tension, the initially hemispherical droplet deforms into a Taylor cone and ejects a charged polymer jet toward the grounded collector. During flight, electrostatic acceleration and Coulombic repulsion among like charges promote extensive jet stretching and thinning, while viscoelastic resistance and aerodynamic drag influence jet deformation and whipping behavior. Although gravitational force may be included in a complete force balance, its contribution during conventional electrospinning is generally much smaller than that of the electric-field-driven acceleration. Thus, the balance among electrostatic force, Coulombic repulsion, surface tension, viscoelastic resistance, and aerodynamic drag governs jet initiation and subsequent fiber formation (Figure 1b).

Manipulation of the collector geometry altered the local electric-field distribution and thereby controlled the direction of fiber deposition. For uniaxially aligned fibers, a taping-assisted collector configuration was used to restrict the available deposition region and preferentially guide fibers across the defined collector gap (Figure 1b(i)). In the taping-assisted collector configuration, fibers were preferentially deposited and aligned on the taped regions, whereas fibers collected on the adjacent untaped regions exhibited a comparatively random orientation. This spatially selective deposition enabled the formation of distinct aligned and random fibrous regions within a single electrospinning process. For radial fiber organization, a ring–pin collector consisting of a peripheral conductive ring and a central conductive pin was employed (Figure 1b(ii)). Ring–pin electrode configurations have previously been demonstrated to divide the local electric-field trajectories between the central point electrode and surrounding ring, directing charged fibers across the intervening gap and producing radial alignment. Fibers deposited between the central pin and peripheral ring followed radial trajectories extending from the center toward the outer boundary, providing a straightforward strategy for generating the radial tie-fiber-like architecture required for the meniscus-mimetic design.

The effectiveness of collector-mediated topographical control was subsequently verified by SEM as shown in Figure 1c. At the boundary generated using the taping-assisted collector, a clear transition from randomly distributed fibers to highly directionally organized fibers was observed (Figure 1c(i)), demonstrating spatial control of fiber orientation within the same fabricated scaffold. In contrast, the ring–pin collector produced fibers extending along radial trajectories between the central and peripheral regions (Figure 1c(ii)), confirming the successful formation of a radial fibrous architecture. This collector-dependent organization is consistent with the principle that modulation of collector geometry and local electric-field distribution can direct electrospun fiber deposition without altering the fundamental electrospinning process. To further visualize differences in local fiber orientation, SEM images were analyzed using the Orientation J plugin in ImageJ, and orientation-dependent false-color maps were generated as presented in Figure 1d. The random region displayed a heterogeneous distribution of orientation-associated colors, reflecting the absence of a dominant fiber direction, whereas the aligned region exhibited a comparatively uniform orientation pattern. Similarly, the radial scaffold showed a spatially varying orientation field corresponding to the radial trajectories of the fibers. These orientation maps provided a qualitative spatial representation of fiber directionality and complemented the morphological observations obtained by SEM.

Finally, the internal structure of the electrospun fibers and incorporation of functional nanomaterials were examined by TEM. Pristine PVDF fibers exhibited a comparatively homogeneous internal morphology without discernible particulate inclusions (Figure 1e). In contrast, TEM images of the PVDF/BTO/MWCNT composite fibers revealed BTO nanoparticles together with MWCNT structures embedded within the PVDF fiber matrix (Figure 1f), confirming the incorporation of both functional components into the electrospun fibers. Together, these findings demonstrate that the fabrication strategy enabled independent control over fiber architecture—random, aligned, and radial—while simultaneously permitting incorporation of BTO and MWCNTs into the PVDF matrix. This combination established a structurally diverse scaffold platform for subsequently determining how meniscus-mimetic topography and material composition influence cellular organization and phenotype.

### 3.2. Structural Characterization of Topographically and Compositionally Distinct Nanofibrous Scaffolds

**Figure 2:**
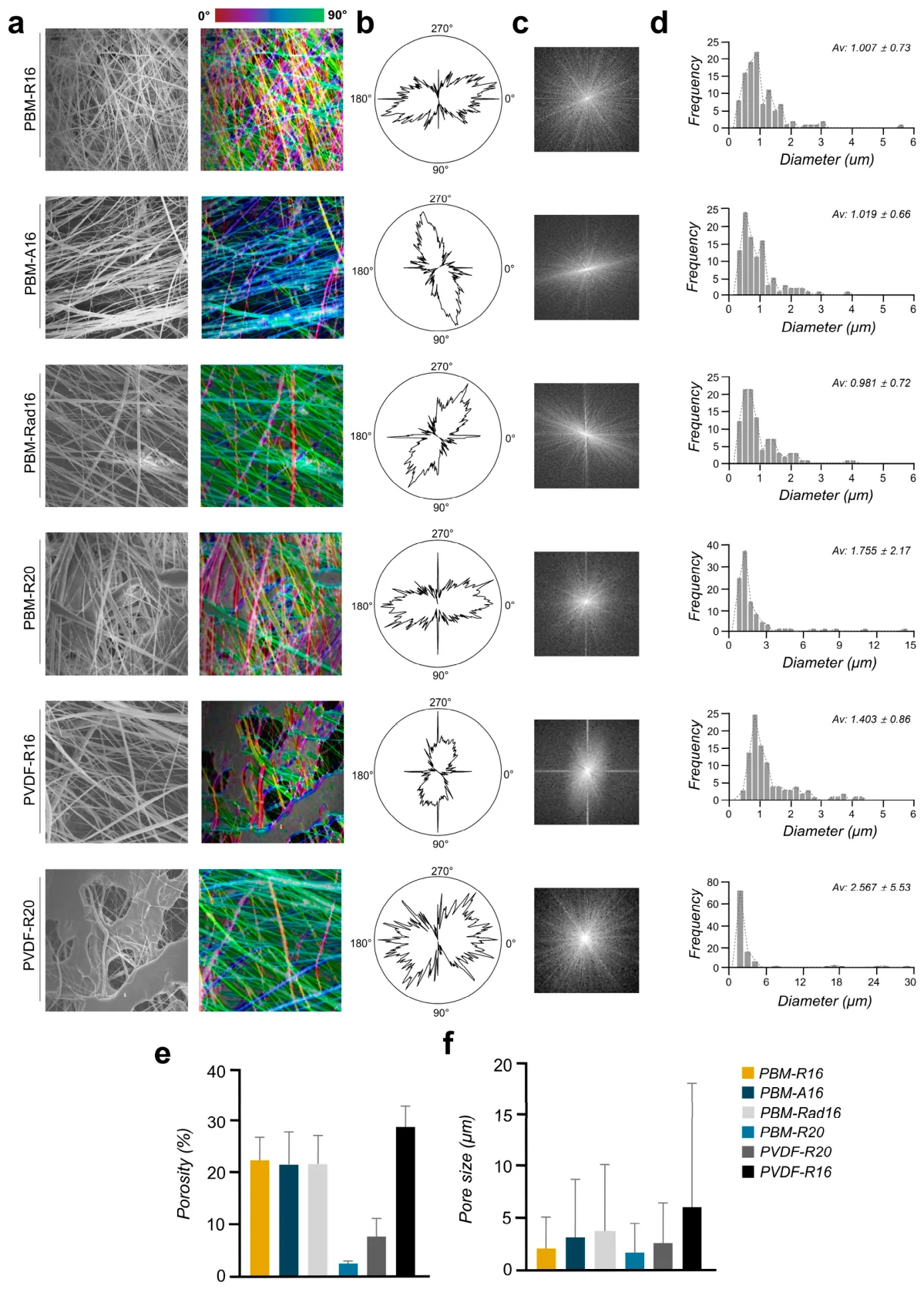
Quantitative characterization of fiber morphology, orientation, and porous architecture of PVDF-based nanofibrous scaffolds. (**a**) Representative high-magnification SEM images and corresponding Orientation J false-color orientation maps of PBM-R16, PBM-A16, PBM-Rad16, PBM-R20, PVDF-R16, and PVDF-R20 scaffolds. (**b**) Angular fiber-orientation profiles over 0–360° derived from FFT analysis of representative SEM images. (**c**) Representative grayscale two-dimensional FFT patterns corresponding to the SEM images, visualizing the degree and directionality of spatial anisotropy within each fibrous architecture. (**d**) Quantitative comparison of fiber diameter among the six scaffold groups. (**e**) Quantitative comparison of scaffold porosity. (**f**) Quantitative comparison of pore size. FFT analyses were performed using representative SEM images selected from three independently fabricated scaffolds per group (*n* = 3). Porosity and pore size were quantified using approximately 100 measurements per group. Data are presented as mean ± SD.

Following establishment of the collector configurations for controlling fiber deposition, the morphological and structural characteristics of the six scaffold groups were systematically evaluated (Figure 2). The scaffolds were designated as PBM-R16 (PVDF/BTO/MWCNT, random, 16 wt% PVDF), PBM-A16 (PVDF/BTO/MWCNT, aligned, 16 wt% PVDF), PBM-Rad16 (PVDF/BTO/MWCNT, radial, 16 wt% PVDF), PBM-R20 (PVDF/BTO/MWCNT, random, 20 wt% PVDF), PVDF-R16 (pristine PVDF, random, 16 wt%), and PVDF-R20 (pristine PVDF, random, 20 wt%). This experimental design enabled the effects of fiber topography, polymer concentration, and incorporation of BTO/MWCNTs to be examined within a common electrospun PVDF-based platform.

Higher-magnification SEM images revealed distinct differences in fiber organization and morphology among the six scaffold groups as shown in Figure 2a. PBM-R16 exhibited an interconnected fibrous network without a preferential orientation, whereas PBM-A16 showed fibers predominantly arranged along a common axis. PBM-Rad16 displayed locally directional fibers consistent with the radial architecture generated using the ring–pin collector. Importantly, despite these differences in orientation, the three PBM scaffolds fabricated from the same 16 wt% formulation retained generally comparable fiber morphology, indicating that collector-mediated topographical control could modify fiber directionality without substantially altering the basic fiber-forming characteristics of the electrospinning solution. The corresponding OrientationJ false-color maps further visualized these architectural differences, with heterogeneous orientation distributions in the random scaffolds and more spatially coherent orientation patterns in the aligned and radial configurations.

Fiber organization was further examined by fast Fourier transform (FFT) analysis of the SEM images (Figure 2b,c). The angular FFT profiles plotted over 0–360° provided a quantitative representation of orientation-dependent spatial frequencies within each scaffold. Random fibrous networks exhibited comparatively broad angular distributions, consistent with multidirectional fiber organization, whereas the aligned scaffold produced pronounced orientation-dependent peaks reflecting preferential fiber alignment. In the radial scaffold, the orientation distribution reflected the spatially varying fiber directions inherent to the radial geometry; therefore, its FFT characteristics were interpreted in conjunction with the local regions analyzed rather than as a single global uniaxial direction. The corresponding grayscale two-dimensional FFT patterns provided an additional visual representation of the frequency-domain anisotropy of each fibrous architecture (Figure 2c). Together with the Orientation J maps, these analyses confirmed that the collector configurations produced distinguishable random, aligned, and radial topographical environments.

In addition to fiber orientation, substantial differences in fiber dimensions were observed according to scaffold composition and PVDF concentration (Figure 2d). Among PBM-R16, PBM-A16, and PBM-Rad16, fiber diameters remained relatively comparable despite their markedly different directional architectures. This observation further indicates that the collector configurations primarily controlled the spatial organization of fiber deposition, while the characteristic fiber dimensions were largely maintained when the electrospinning formulation and processing conditions were held constant. In contrast, increasing the PVDF concentration from 16 to 20 wt% resulted in an overall tendency toward larger fiber dimensions, accompanied in the present experimental system by increasingly irregular fiber morphology. Polymer concentration is an important determinant of electrospun fiber dimensions because changes in concentration alter solution viscosity and polymer-chain entanglement and thereby affect jet stretching and fiber formation. Previous studies have likewise identified polymer concentration as a major determinant of electrospun PVDF fiber diameter, although the exact morphology–concentration relationship depends strongly on the solvent system and other electrospinning conditions. Accordingly, the morphological deterioration observed at 20 wt% in the present system should be interpreted as a formulation-specific response under the electrospinning conditions used here rather than as a universal consequence of increasing PVDF concentration.

A composition-dependent difference was also evident at 16 wt%, with PVDF-R16 exhibiting a larger fiber diameter than PBM-R16. Because both scaffolds were fabricated using the same polymer concentration and electrospinning parameters, this difference suggests that the incorporation of BTO nanoparticles and MWCNTs influenced the electrospinning behavior and resulting fiber morphology. In particular, MWCNTs are electrically conductive nanomaterials that may modify the electrical properties of polymer-based electrospinning solutions. Increased charge transport within the electrospinning jet can enhance electrostatic stretching and jet thinning, potentially contributing to the formation of fibers with smaller diameters. Accordingly, the smaller fiber diameter observed in PBM-R16 compared with PVDF-R16 may be partially associated with the conductive characteristics of the incorporated MWCNTs. However, the morphological effects of MWCNT incorporation are not governed exclusively by electrical conductivity. MWCNTs may also alter solution viscosity, polymer-chain interactions, and nanofiller dispersion, which can influence jet stability, fiber uniformity, and the formation of morphological irregularities. Furthermore, the simultaneous incorporation of BTO nanoparticles may introduce additional changes in solution properties and fiber formation. Therefore, the observed reduction in fiber diameter should be interpreted as a combined effect of nanofiller incorporation rather than being attributed solely to MWCNT-induced conductivity changes.

Although solution viscosity and electrical conductivity were not directly measured in the present study, the observed differences in fiber diameter and morphology suggest that nanofiller incorporation altered the electrospinning process. Further investigation of the individual contributions of BTO and MWCNTs, together with direct measurements of solution properties, would be necessary to clarify the mechanisms underlying these morphological changes. The differences in fiber formation were further reflected in scaffold porosity and pore size (Figure 2e,f). Among the three 16 wt% PBM scaffolds, PBM-R16, PBM-A16, and PBM-Rad16 exhibited comparable overall porosities, despite their different fiber orientations. Thus, the collector-induced transition from random to aligned or radial organization did not substantially alter the overall void fraction of the fibrous matrices under the present fabrication conditions. This is particularly important for the subsequent biological analyses because it allows cellular responses among these three groups to be interpreted primarily in relation to differences in fiber organization rather than major differences in total scaffold porosity. By comparison, PBM-R20 exhibited the lowest porosity, consistent with the formation of thicker fibers and a denser fibrous architecture at the higher PVDF concentration. Interestingly, PVDF-R20 showed a markedly different structural response. This scaffold exhibited the most pronounced disruption of uniform fiber formation and, concurrently, relatively high porosity and enlarged pore size (Figure 2e,f). Rather than indicating an improved porous network, these increased values appeared to arise from reduced or spatially heterogeneous fiber deposition, which generated larger interfiber voids. Thus, porosity alone did not fully describe the quality or organization of the electrospun network; scaffolds with similarly high porosity could possess fundamentally different fiber distributions and pore architectures. Consistent with this interpretation, the pore-size analysis showed that PVDF-R20 contained the largest interfiber pores, corresponding to its irregular and sparsely distributed fibrous morphology. Electrospun membranes inherently possess interconnected void spaces whose dimensions depend on fiber diameter, fiber packing density, deposition amount, and spatial organization. Therefore, the simultaneous increase in porosity and pore size observed for PVDF-R20 is most plausibly interpreted as a consequence of the reduced packing density and heterogeneous deposition observed morphologically, rather than simply as an effect of fiber diameter.

These results demonstrate that fiber orientation could be modulated independently of major changes in fiber diameter and overall porosity among the 16 wt% PBM scaffolds, whereas changes in PVDF concentration and material composition substantially affected fiber formation and the resulting pore architecture. These quantitatively distinct structural features—including fiber orientation, fiber diameter, porosity, and pore size—therefore established a multidimensional set of scaffold descriptors for subsequent evaluation of topography-dependent cellular responses and machine learning-assisted structure–response relationships.

### 3.3. Experimental Design for Quantitative Assessment of Scaffold-Dependent Cellular Responses

Following structural characterization, nine experimental conditions were established for quantitative assessment of scaffold-dependent cellular responses (Table 1). Samples 1–6 comprised the six nanofibrous scaffold groups: PBM-R16, PBM-A16, PBM-Rad16, PBM-R20, PVDF-R16, and PVDF-R20. Each scaffold was prepared as a 12-mm-diameter specimen, placed individually in a 48-well culture plate, and seeded with 2 × 10^4^ hBMSCs per scaffold. An identical initial seeding number was maintained across all scaffold groups to enable direct comparison of subsequent cellular responses, including cellularity, nuclear morphology and orientation, cytoskeletal organization, and SOX9-associated phenotype, while minimizing variation attributable to differences in the initial cell number.

Nonfibrous tissue culture plate (TCP) conditions were additionally included as reference substrates (Samples 7–9). In contrast to cells cultured on nanofibrous scaffolds, where fiber architecture provides physical constraints and directional contact-guidance cues, hBMSCs cultured directly on TCP exhibited extensive planar spreading. When TCP was seeded with 2 × 10^4^ cells/well, corresponding to the initial cell number used for the scaffold groups, extensive cell spreading resulted in densely occupied regions and frequent spatial overlap among neighboring cells. Such overlap can complicate individual-cell segmentation and subsequent extraction of image-derived morphological and organizational features, which were central to the quantitative imaging workflow used in this study.

To account for this effect, three TCP reference conditions with different initial seeding numbers—5 × 10^3^, 1 × 10^4^, and 2 × 10^4^ cells/well—were included and designated TCP-5K, TCP-10K, and TCP-20K, respectively. These conditions provided nonfibrous reference images spanning relatively low to high cellular occupancy, thereby allowing the image-analysis pipeline to evaluate cellular features across a broader range of cell distributions. Importantly, TCP-5K and TCP-10K were included as cell-occupancy reference conditions for quantitative imaging and computational analysis, rather than as direct biological controls for the scaffold groups. In contrast, TCP-20K, which received the same initial number of cells as Samples 1–6, served as the seeding-number-matched nonfibrous reference.

This distinction was maintained throughout the subsequent quantitative and machine learning-assisted analyses [41]. Biological comparisons of scaffold-dependent responses were primarily based on Samples 1–6, for which the initial seeding number was fixed at 2 × 10^4^ cells per scaffold, whereas the TCP series provided additional reference conditions for evaluating image-derived features across different levels of cellular occupancy. This design minimized potential confounding between initial cell number and scaffold-dependent effects while providing a broader reference range for object-level segmentation and computational characterization of cellular responses.

### 3.4. Deep Learning-Assisted Quantification of Nuclear Morphology and Organization

**Figure 3:**
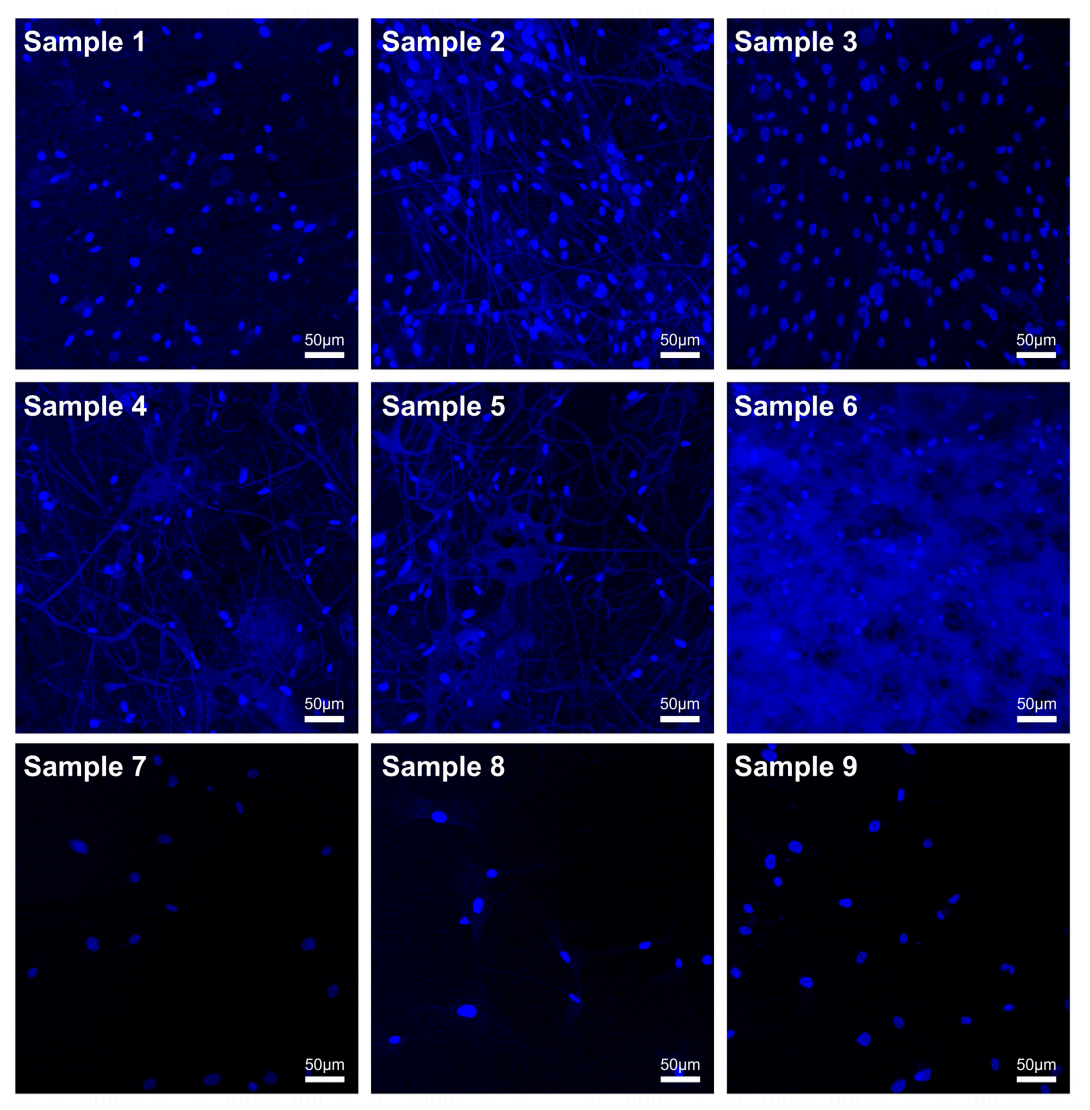
Representative DAPI fluorescence images of hBMSCs cultured on nanofibrous scaffolds and nonfibrous TCP reference substrates. Images are representative of three independent experiments (*n* = 3).

Representative DAPI fluorescence images of hBMSCs cultured under the nine experimental conditions are shown in Figure 3. Samples 1–6 corresponded to the nanofibrous scaffold groups PBM-R16, PBM-A16, PBM-Rad16, PBM-R20, PVDF-R16, and PVDF-R20, respectively, whereas Samples 7–9 represented the TCP reference conditions initially seeded with 5 × 10^3^, 1 × 10^4^, and 2 × 10^4^ cells/well, respectively. Visual inspection revealed distinct differences in the abundance and spatial distribution of DAPI-positive nuclei among the experimental conditions. The nanofibrous scaffold groups generally exhibited abundant DAPI-positive nuclei, although their spatial distributions varied among the different scaffold formulations and fiber architectures. In contrast, the TCP reference groups showed comparatively lower nuclear occupancy within the representative imaging fields, irrespective of their different initial seeding numbers. Notably, even the TCP condition seeded with 2 × 10^4^ cells/well exhibited fewer nuclei within the representative fields than were generally observed in the nanofibrous scaffold groups seeded with the same initial number of cells.

These observations suggest that the cellular distribution established on the nonfibrous TCP surface differed substantially from that observed on the nanofibrous matrices. However, because Figure 3 presents representative microscopic fields rather than measurements of the entire culture surface, the lower number of nuclei observed in the TCP images cannot by itself be attributed to differences in cell proliferation, survival, or attachment. The observed nuclear abundance may additionally be influenced by substrate-dependent cell distribution, migration, retention, and local sampling within the field of view. Accordingly, visual differences among the representative DAPI images were treated as qualitative observations, and subsequent comparisons were based on quantitative image-derived measurements.

To objectively characterize nuclear abundance and morphology, DAPI-positive nuclei were analyzed using a deep learning-assisted image segmentation workflow based primarily on the pretrained Cellpose nuclei model [42,43]. Cellpose-based instance segmentation identified individual nuclei and converted the fluorescence images into labeled nuclear masks, enabling quantitative measurements to be extracted at the individual-object level. For predefined images in which the pretrained model showed inadequate segmentation, including missed nuclei or inappropriate segmentation of background regions, a classical image-processing procedure incorporating Gaussian smoothing, Otsu thresholding, and connected-component analysis was applied as a fallback approach [43]. The resulting labeled masks were subsequently used to determine the number of detected nuclei and to extract morphological and orientation-related descriptors from individual nuclear objects [37].

Across the analyzed nanofibrous scaffold images, the number of detected nuclei averaged 137.04 ± 50.10 nuclei per image, with values ranging from 58 to 231 (Table 2). Because Samples 1–6 were uniformly seeded with 2 × 10^4^ hBMSCs per scaffold, differences in image-level nuclear counts could not be attributed to differences in the initial seeding number among these groups. Instead, these variations reflected differences in the number of nuclei present within the analyzed fields at the imaging endpoint. Nevertheless, nuclear count was interpreted as a measure of local cellular occupancy rather than direct evidence of cell proliferation, because attachment, survival, migration, spatial distribution, and field-to-field heterogeneity may all influence the number of cells detected within a microscopic field.

The segmented nuclear masks further enabled quantitative characterization of nuclear morphology. Nuclear elongation was calculated as the ratio of the major-axis length to the minor-axis length of each segmented nucleus. Across the analyzed scaffold images, the image-level median nuclear elongation averaged 1.496 ± 0.073, with values ranging from 1.388 to 1.680. These differences indicate that nuclear shape varied among the scaffold conditions, suggesting differential morphological adaptation of hBMSCs to their surrounding fibrous microenvironments. In particular, anisotropic topographical cues can influence cellular organization and cytoskeletal tension, which may subsequently be reflected in nuclear deformation and elongation. Nuclear elongation was therefore retained as a quantitative descriptor of scaffold-dependent nuclear morphology. However, it was not considered equivalent to whole-cell elongation or cytoskeletal organization, which were independently evaluated using F-actin imaging.

Directional nuclear organization was further characterized from the principal axis of each segmented nucleus. Nuclear orientation was represented as an axial angle ranging from 0° to 180°, because orientations separated by 180° describe the same physical axis. Accordingly, doubled-angle circular statistics were applied to appropriately analyze these axial orientation data. The nuclear alignment index was calculated as the magnitude of the mean doubled-angle orientation vector and ranged from 0, representing an isotropic distribution of nuclear orientations, to 1, representing complete directional alignment along a common axis. Across the analyzed images, the nuclear alignment index averaged 0.266 ± 0.168, with values ranging from 0.037 to 0.607, indicating substantial differences in the degree of directional nuclear organization among the experimental conditions.

The interpretation of nuclear orientation was further considered in the context of the underlying scaffold architecture. Uniaxially aligned scaffolds provide a relatively uniform reference direction across the imaging field, whereas radial scaffolds exhibit spatially varying local fiber orientations relative to the center of the radial architecture. Consequently, a single absolute orientation angle does not provide an equivalent measure of topographical responsiveness across random, aligned, and radial architectures. Nuclear orientation parameters were therefore interpreted primarily as descriptors of directional organization and were subsequently evaluated together with scaffold fiber orientation and F-actin-derived cellular alignment. This combined analysis enabled a more direct assessment of the correspondence between the engineered fiber architecture and cellular organization.

Nuclear area was additionally extracted from the segmented masks as a complementary descriptor of nuclear morphology. The area of each segmented nucleus was calculated from the number of pixels contained within the corresponding mask and converted to physical units using the image-scale calibration. Because accurate interpretation of nuclear area depends directly on the pixel-to-micrometer conversion, the final calibrated values were incorporated only after verification of the image scale. Nuclear area was subsequently considered together with nuclear count, elongation, and alignment to establish a multidimensional description of nuclear responses to the different scaffold environments.

Overall, the DAPI-based analysis demonstrated that the experimental conditions were associated with distinct patterns of local cellular occupancy, nuclear morphology, and directional nuclear organization. Importantly, the analysis progressed beyond qualitative fluorescence-image comparison by employing a pretrained deep learning-based segmentation framework to generate reproducible object-level nuclear descriptors. Cellpose was used specifically for image segmentation and feature extraction and was therefore distinguished from the predictive machine learning models employed in the subsequent scaffold–response analysis [35,44]. The resulting nuclear features were subsequently integrated with F-actin-derived cytoskeletal parameters and SOX9-associated molecular responses to construct the multidimensional biological dataset used for machine learning-assisted evaluation of scaffold–cell relationships [45].

### 3.5. Scaffold Topography Directs Cytoskeletal Organization and Cellular Alignment

**Figure 4:**
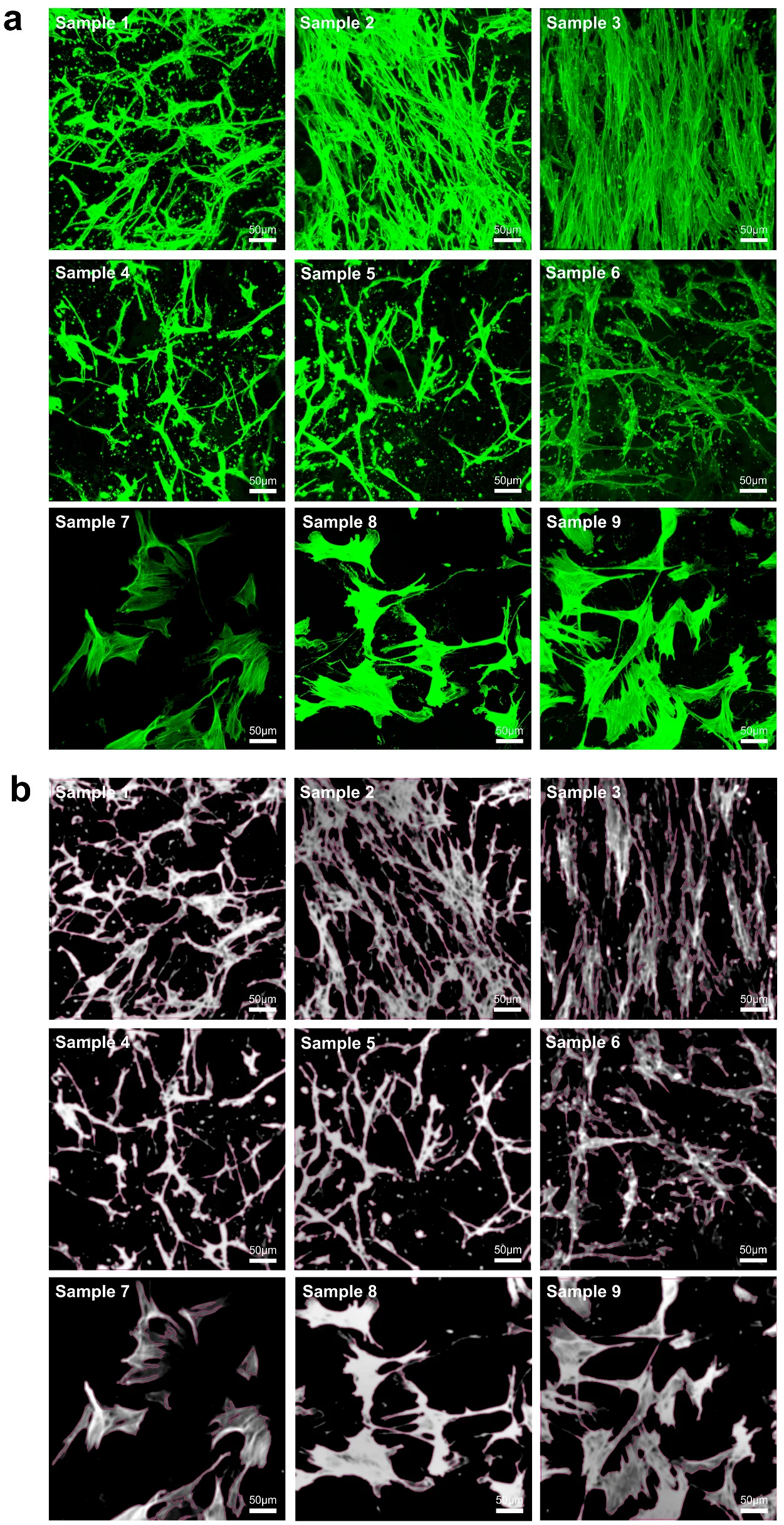
Topography-dependent cytoskeletal organization of hBMSCs cultured on nanofibrous scaffolds and TCP reference substrates. (**a**) Representative F-actin fluorescence images of hBMSCs cultured under Samples 1–9. (**b**) Representative processed F-actin images of Samples 1–9 showing background-corrected F-actin signals with Otsu-derived positive-mask contours used for quantitative assessment of F-actin coverage. Images are representative of three independent experiments (*n* = 3).

Following the nuclear-level analysis, F-actin staining was performed to evaluate whole-cell morphology and cytoskeletal organization in response to the different scaffold environments. Representative F-actin fluorescence images of Samples 1–9 are shown in Figure 4a. Marked differences in cellular morphology and organization were observed between the nanofibrous scaffold groups and the nonfibrous TCP reference conditions. Cells cultured on the nanofibrous scaffolds (Samples 1–6) generally exhibited an elongated and slender morphology, with F-actin-rich cytoskeletal structures extending preferentially along the underlying fibrous architecture. In contrast, cells cultured on TCP (Samples 7–9) displayed a broader and more extensively spread morphology without an apparent preferential direction, indicating fundamentally different cytoskeletal organization on planar and fibrous substrates.

The influence of fiber topography was particularly evident among PBM-R16, PBM-A16, and PBM-Rad16, which shared the same material composition and PVDF concentration but differed in fiber architecture. Cells cultured on PBM-R16 exhibited multidirectional cytoskeletal organization consistent with the randomly distributed fiber network. In contrast, PBM-A16 induced pronounced cellular elongation and preferential organization along a common axis, closely corresponding to the uniaxially aligned fiber architecture. PBM-Rad16 produced a distinctly different spatial organization, with elongated cellular structures following locally varying radial trajectories. Thus, altering fiber orientation while maintaining the same PBM formulation generated clearly distinguishable cellular morphologies, supporting a dominant role of scaffold topography in directing cytoskeletal organization through contact guidance.

Differences were also observed among PBM-R20, PVDF-R16, and PVDF-R20, indicating that scaffold composition and fiber morphology additionally influenced cellular organization. However, these differences were less pronounced than those produced by changing fiber architecture among the 16 wt% PBM scaffolds. In particular, variations in PVDF concentration and incorporation of BTO/MWCNTs altered fiber diameter, porosity, pore size, and overall fibrous morphology, as demonstrated in Figure 2, and these structural differences may contribute to variations in cellular spreading and cytoskeletal organization. Nevertheless, the visually strongest changes in cellular directionality were associated with the transition among random, aligned, and radial fiber architectures, suggesting that fiber orientation represented a major topographical cue governing directional cellular organization in the present scaffold system.

To quantitatively characterize these observations, the F-actin fluorescence images were processed using a pixel-based image-analysis workflow without individual-cell segmentation (Figure 4b). Fluorescence images were normalized and corrected for slowly varying background intensity, after which Otsu thresholding was applied to distinguish F-actin-associated fluorescence from background signal [46]. Pixels above the automatically determined threshold were classified as F-actin-positive, and the resulting binary masks were refined to remove small isolated objects and edge-related artifacts. F-actin coverage was calculated as the proportion of F-actin-positive pixels relative to the total analyzed image area:

(1) $$F-actin\;coverage\;\left(\%\right)=\frac{N_{F-actin-positive}}{N_{total\;image}}\times 100$$

Accordingly, F-actin coverage represented the spatial extent of detectable cytoskeletal fluorescence rather than the proportion of individually segmented cells. Local cytoskeletal orientation was subsequently extracted from the background-corrected F-actin images using structure-tensor-based analysis [46]. A custom Python-based computational pipeline was subsequently applied to compare cellular organization with the underlying scaffold architecture. Scaffold fiber orientation was independently extracted from SEM images using fast Fourier transform (FFT)-based analysis, whereas local F-actin orientation was quantified using structure-tensor-based analysis [47]. Structure-tensor analysis enabled the dominant local orientation of the cytoskeletal signal to be determined without requiring individual cell boundaries to be explicitly segmented, making it particularly suitable for analyzing densely distributed and interconnected F-actin structures [48]. Quantitative analysis of the F-actin images further demonstrated substantial variation in cytoskeletal organization across the scaffold conditions (Table 3). The mean F-actin local coherence was 0.513 ± 0.070, with values ranging from 0.394 to 0.695, indicating differences in the consistency of local cytoskeletal orientation among the analyzed images. The F-actin alignment index, which describes the degree of global directional organization on a scale from 0 (isotropic) to 1 (highly aligned), averaged 0.394 ± 0.282 and ranged from 0.043 to 0.934. The broad range of alignment indices was consistent with the intentionally distinct fiber architectures, encompassing random, uniaxially aligned, and radially organized topographies. In addition, F-actin coverage averaged 26.61 ± 5.78%, with values ranging from 16.85 to 38.75%, demonstrating variation in the fraction of the analyzed area occupied by F-actin-positive cytoskeletal structures. These quantitative descriptors confirmed that the scaffold conditions differed not only in the apparent morphology of the cells but also in the degree, consistency, and spatial extent of cytoskeletal organization [49].

The scaffold- and cell-derived orientation distributions were subsequently compared to determine scaffold–cell alignment correspondence, which represented the extent to which cellular organization followed the underlying fiber architecture. This approach was important because conventional measurements of cellular alignment alone do not necessarily indicate whether the observed orientation originates from scaffold-mediated contact guidance. By directly relating the orientation of the F-actin cytoskeleton to that of the corresponding scaffold fibers, the analysis provided a quantitative link between the engineered topographical cue and the resulting cellular organization.

This distinction was particularly relevant for the radial scaffold. Unlike a uniaxially aligned scaffold, for which fiber and cell orientation can be described relative to a single global axis, the orientation of radial fibers changes continuously as a function of spatial position. Consequently, global orientation measurements can underestimate organization within radial architectures. Local orientation analysis allowed the spatially varying F-actin trajectories to be compared with the corresponding local fiber directions, providing a more appropriate measure of cellular conformity to the radial topography.

The TCP groups exhibited a distinctly different F-actin organization. Although differences in initial seeding number resulted in some variation in cellular occupancy among the TCP conditions, cells on the planar TCP surface generally displayed broad cytoplasmic spreading and heterogeneous F-actin orientations without the directional organization characteristic of the nanofibrous scaffold groups. Thus, differences between the scaffold and TCP groups extended beyond cell number and were evident in the overall architecture of the actin cytoskeleton. These findings further demonstrate that the fibrous microenvironment provided physical guidance cues that were absent from the conventional planar culture surface. The F-actin analysis demonstrated that the engineered nanofibrous architectures regulated not only cellular spreading but also the direction and spatial organization of the cytoskeleton. The close correspondence between the distinct random, aligned, and radial architectures and their associated cellular morphologies, particularly among the composition-matched PBM-R16, PBM-A16, and PBM-Rad16 groups, supports topography-mediated contact guidance as a major determinant of cellular organization. The quantitative parameters derived from this analysis, including F-actin coverage, cellular orientation, and scaffold–cell alignment correspondence, were subsequently incorporated with the DAPI-derived nuclear descriptors and SOX9-associated responses to establish the multidimensional cellular dataset used for downstream machine learning-assisted analysis.

### 3.6. Quantitative Assessment of Nuclear SOX9 Expression and Meniscus-Associated Phenotype

Following characterization of nuclear morphology and cytoskeletal organization, SOX9 expression was evaluated to determine whether the distinct scaffold environments were associated with differences in a chondrogenic phenotype of hBMSCs. SOX9 is a transcription factor closely associated with chondrogenic lineage regulation and extracellular matrix-related gene expression and was therefore selected as a molecular readout complementary to the morphological and organizational parameters obtained from DAPI and F-actin analyses. Importantly, SOX9 expression was interpreted in the present study as a chondrogenic phenotype-associated indicator rather than a standalone measure of complete meniscal differentiation.

**Figure 5:**
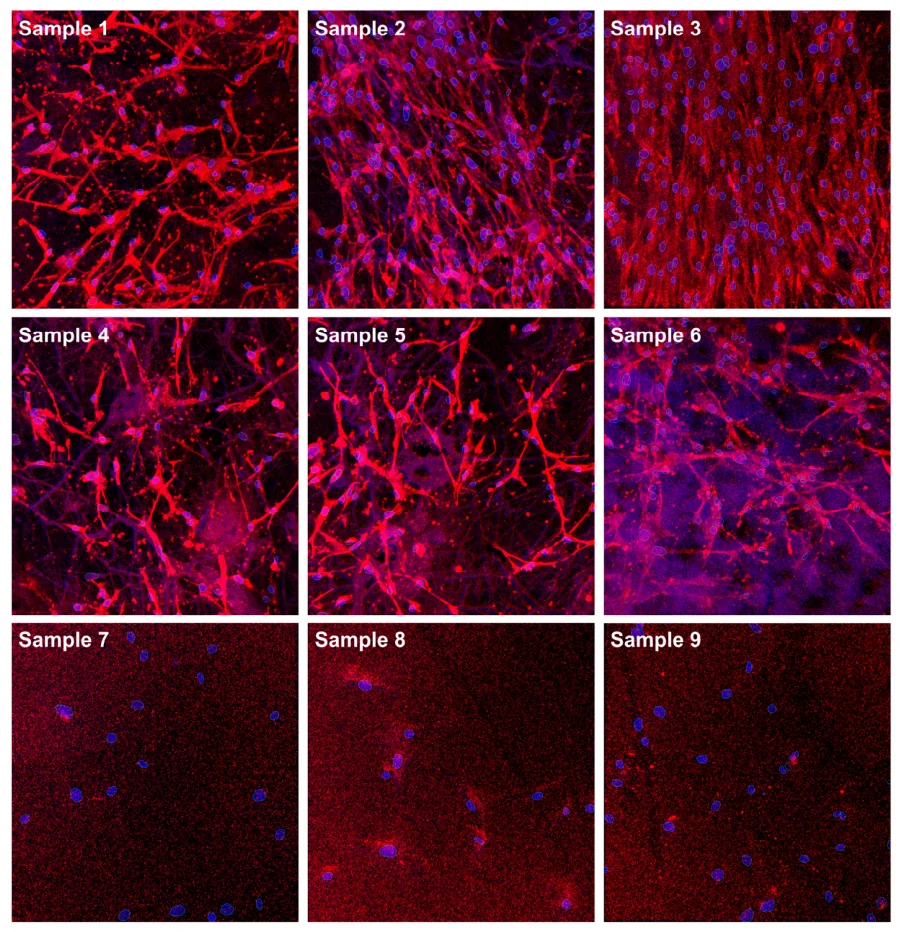
Nuclear SOX9 expression in hBMSCs cultured on nanofibrous scaffolds and nonfibrous TCP reference substrates. Images are representative of three independent experiments (*n* = 3).

Representative SOX9 immunofluorescence images of Samples 1–9 are shown in Figure 5. Samples 1–6 corresponded to PBM-R16, PBM-A16, PBM-Rad16, PBM-R20, PVDF-R16, and PVDF-R20, respectively, whereas Samples 7–9 represented the TCP reference conditions with different initial seeding numbers. Qualitative differences in SOX9 fluorescence intensity and the proportion of SOX9-expressing nuclei were apparent among the experimental conditions. However, because differences in cell number, spatial distribution, and local background fluorescence can substantially influence field-level fluorescence intensity, SOX9 expression was not evaluated solely from the overall fluorescence intensity of representative images. Instead, a nucleus-resolved quantitative analysis was performed to evaluate SOX9 signals specifically within DAPI-defined nuclear regions.

For quantitative analysis, DAPI-derived nuclear masks were used to define individual nuclear regions of interest, and SOX9 fluorescence intensity was measured within each nuclear mask after background correction. To account for local background fluorescence, an annular background region was defined around each DAPI-derived nucleus while excluding neighboring nuclear regions. The median SOX9 intensity within this surrounding region was used as the corresponding local background estimate. The background-corrected nuclear SOX9 intensity was calculated by subtracting the corresponding local background intensity from the mean SOX9 intensity measured within each DAPI-defined nucleus:

(2) $$I_{corrected}=I_{nucleus}-I_{background}$$

where $I_{nucleus}$ represents the mean SOX9 intensity within the DAPI nuclear mask and $I_{background}$ represents the corresponding local background median.

This nucleus-resolved approach reduced the influence of differences in cell number and non-nuclear fluorescence on SOX9 quantification and enabled SOX9 expression to be assessed at the individual-nucleus level. To classify SOX9-positive nuclei without manually assigning group-specific thresholds, a single global Otsu threshold was derived from the distribution of background-corrected nuclear SOX9 fluorescence intensities and applied consistently across the experimental dataset [50]. The proportion of SOX9-positive nuclei was then calculated as the percentage of segmented nuclei with background-corrected SOX9 intensities exceeding this threshold.

Accordingly, the percentage of SOX9-positive nuclei was calculated as:

(3) $$SOX9-positive\;nuclei\;\left(\%\right)=\frac{N_{SOX9-positive\;nuclei}}{N_{total\;DAPI\;nuclei}}\times 100$$

Quantitative analysis revealed considerable variation in SOX9-associated parameters across the analyzed conditions (Table 4). The proportion of SOX9-positive nuclei averaged 29.30 ± 14.24%, with values ranging from 3.90% to 58.73%, demonstrating a broad range of nuclear SOX9 positivity among the analyzed images. The median background-corrected nuclear SOX9 intensity was 5.871 ± 4.911 arbitrary units (AU) and ranged from −0.473 to 19.997 AU. The occurrence of slightly negative background-corrected values reflects cases in which the estimated local background intensity marginally exceeded the nuclear fluorescence signal and should therefore be interpreted as an absence of detectable signal above background rather than as negative biological expression.

The heterogeneity of nuclear SOX9 fluorescence was additionally characterized using the interquartile range (IQR) of background-corrected nuclear intensities. The mean IQR was 21.220 ± 11.695 AU, ranging from 7.488 to 51.160 AU, indicating substantial variation in the distribution of nuclear SOX9 signals among the analyzed conditions. Because fluorescence intensity measurements may also be affected by residual image-to-image background variation, complementary normalized measures were calculated. The median nuclear-to-background SOX9 intensity ratio was 1.055 ± 0.039, with values ranging from 0.995 to 1.124, whereas the median robust SOX9 signal-to-noise ratio was 0.163 ± 0.106, ranging from −0.018 to 0.354. Together, these complementary parameters characterized both the magnitude and detectability of nuclear SOX9 fluorescence relative to the surrounding image background.

The use of multiple SOX9-derived descriptors was particularly important because absolute fluorescence intensity alone may be influenced by technical factors, even when image acquisition settings are maintained consistently. The SOX9-positive nuclear fraction provided a cell-normalized measure of the prevalence of SOX9-associated responses, whereas background-corrected intensity and nuclear-to-background measurements provided complementary information regarding the relative magnitude of nuclear SOX9 signals. Accordingly, interpretation was based on the collective pattern of these measurements rather than on a single fluorescence-intensity parameter.

In the context of the preceding DAPI and F-actin analyses, these SOX9 measurements provided a molecular dimension complementary to cellular occupancy, nuclear morphology, and cytoskeletal organization. The engineered scaffolds therefore generated a multidimensional spectrum of cellular responses encompassing cell quantity, morphological and directional organization, and SOX9-associated phenotype. Because no single parameter adequately represented these different aspects of scaffold-induced cellular behavior, the resulting quantitative descriptors were subsequently integrated into the Meniscus-Mimetic Scaffold Quality Index (MMS-QI). The SOX9-associated domain was assigned greater weight within this composite index to emphasize phenotype-associated responses while retaining complementary information regarding cellularity and scaffold-guided cellular organization. This integrated dataset subsequently served as the biological response space for machine learning-assisted analysis of scaffold structure–response relationships.

### 3.7. Multidimensional Integration of Cellular Responses Using the MMS-QI

The preceding analyses demonstrated that the experimental conditions elicited multidimensional cellular responses involving cellular occupancy, nuclear morphology, cytoskeletal organization, scaffold–cell directional correspondence, and SOX9-associated phenotype. Because these parameters represent distinct aspects of cellular behavior and may not necessarily change in parallel, evaluation based on a single endpoint could incompletely characterize the overall cellular response to each scaffold. The Meniscus-Mimetic Scaffold Quality Index (MMS-QI) was therefore constructed to integrate the image-derived cellular parameters into a composite quantitative framework (Table 5).

The MMS-QI comprised three complementary domains representing cellularity, meniscus organization, and SOX9 expression. The cellularity domain was derived from DAPI-based nuclear abundance, whereas the meniscus organization domain incorporated nuclear morphology and F-actin-based cellular organization. The SOX9 expression domain integrated background-corrected nuclear SOX9 intensity and the proportion of SOX9-positive nuclei. Individual parameters were normalized before integration to minimize differences arising from their original measurement scales. The resulting domain scores were subsequently combined using a predefined weighted scheme to generate the final MMS-QI, with greater emphasis placed on the SOX9 expression domain to reflect its phenotype-associated molecular relevance. MMS-QI values were calculated at the image level and subsequently summarized for each sample as the mean ± SD across four measurements.

The resulting domain profiles demonstrated that the overall MMS-QI was not determined by a single cellular characteristic as presented in Table 5. Among the scaffold groups, Sample 2 (PBM-A16) exhibited an MMS-QI of 64.06 ± 4.11, accompanied by a high cellularity score (0.87 ± 0.10) and a meniscus-organization score of 0.73 ± 0.03. Sample 3 (PBM-Rad16) showed the highest meniscus-organization domain score among the scaffold conditions (0.75 ± 0.02), despite a lower SOX9-expression score (0.30 ± 0.13), resulting in an integrated MMS-QI of 57.01 ± 5.84. Conversely, Sample 5 (PVDF-R16) exhibited the highest mean SOX9-expression domain score among Samples 1–6 (0.47 ± 0.22) but more moderate cellularity and organization scores, yielding an MMS-QI of 57.48 ± 11.30. These contrasting profiles illustrate the rationale for multidimensional integration: a scaffold exhibiting a strong response in one biological domain did not necessarily exhibit correspondingly strong responses across all domains.

The 16 wt% PBM scaffolds further illustrated the influence of topographical architecture on the integrated cellular response. PBM-R16, PBM-A16, and PBM-Rad16 shared the same material formulation but differed in fiber organization, enabling the contribution of topography to be examined with reduced compositional confounding. Their distinct domain profiles indicated that random, aligned, and radial architectures differentially influenced cellular abundance, directional organization, and SOX9-associated responses rather than producing a uniform shift across all cellular endpoints. In particular, the strong organization scores associated with the aligned and radial architectures were consistent with the topography-dependent cytoskeletal responses observed in the F-actin analysis.

Samples 7–9, corresponding to the TCP reference conditions, exhibited substantially lower cellularity domain scores than the scaffold groups, consistent with the lower nuclear occupancy observed in the analyzed DAPI fields. However, these TCP conditions require a different interpretation from Samples 1–6 because TCP-5K and TCP-10K were intentionally established with lower initial seeding numbers. Accordingly, their MMS-QI values were retained as reference profiles within the integrated dataset rather than as direct scaffold-performance comparators. TCP-20K provided the closest seeding-number-matched nonfibrous reference, although differences in substrate geometry and cellular distribution remained intrinsic to the comparison.

Overall, the MMS-QI converted heterogeneous cellular measurements into a common quantitative framework while preserving information from the individual biological domains. Importantly, the index should be interpreted as a study-specific integrative descriptor rather than a universally validated measure of meniscus regeneration or scaffold quality. Its primary purpose in the present study was to provide a consistent multidimensional response variable for comparing cellular response profiles and for subsequent investigation of scaffold structure–response relationships using machine learning.

### 3.8. Machine Learning-Based Prediction of the Meniscus-Mimetic Scaffold Quality Index

Following multidimensional integration of the cellular response parameters into the MMS-QI, machine learning models were evaluated to determine whether the integrated cellular response could be predicted from scaffold-associated features [34,41,51]. Multiple linear and nonlinear regression approaches were compared under a consistent cross-validation framework, including Ridge regression, Elastic Net, Random Forest, Gradient Boosting, Decision Tree, K-nearest neighbors (KNN), support vector regression (SVR), and a convolutional neural network (CNN). In addition, the proposed MMAWE framework was evaluated as an integrated predictive approach. A mean-prediction baseline was included to provide a reference against which the predictive value of the trained models could be assessed.

As summarized in Table 6, substantial differences in predictive performance were observed among the evaluated models. The mean baseline yielded a negative cross-validated R^2^ of −0.342 ± 0.196, with an MSE of 167.290 and MAE of 11.122, indicating poor prediction when scaffold-associated information was not incorporated. In contrast, all trained models substantially reduced prediction error relative to this baseline, demonstrating that the input features contained information associated with variation in the integrated MMS-QI.

Among the conventional regression approaches, Ridge regression showed comparatively strong performance, achieving an R^2^ of 0.723 ± 0.078, an MSE of 34.590, and an MAE of 4.648. Elastic Net exhibited an R^2^ of 0.668 ± 0.120, whereas Gradient Boosting achieved 0.687 ± 0.134. Random Forest and Decision Tree models produced R^2^ values of 0.600 ± 0.130 and 0.593 ± 0.154, respectively. Lower predictive performance was observed for KNN (R^2^ = 0.499 ± 0.143) and SVR (R^2^ = 0.456 ± 0.095). These differences indicate that model architecture and regularization strategy influenced the ability to capture relationships between the input features and integrated cellular response.

The relatively strong performance of Ridge regression suggests that a substantial component of the structure–response relationship could be captured through regularized linear associations. However, the CNN achieved higher predictive performance, with an R^2^ of 0.784 ± 0.099, an MSE of 26.894, and an MAE of 3.907. The improvement obtained with the CNN indicates that additional nonlinear or higher-order relationships may be present within the dataset, although this interpretation should be considered cautiously given the limited number of independent scaffold formulations.

Among all evaluated approaches, MMAWE achieved the highest predictive performance within the present dataset, with an R^2^ of 0.853 ± 0.041, an MSE of 18.362, and an MAE of 3.386. In addition to its higher mean R^2^, MMAWE exhibited a relatively small variation in cross-validated R^2^ compared with the individual nonlinear models, suggesting more consistent performance across the evaluated validation partitions. Relative to the strongest individual model, CNN, MMAWE reduced MSE from 26.894 to 18.362 and MAE from 3.907 to 3.386 while increasing the mean R^2^ from 0.784 to 0.853. These results suggest that integration of complementary predictive information within the MMAWE framework provided a more robust representation of the relationships captured in the present dataset than any single evaluated model.

These predictive results should be interpreted within the scope of the present experimental design. The number of independent scaffold formulations was limited, and multiple observations were derived from the same experimental conditions. Accordingly, cross-validation was performed with separation of scaffold-associated groups to minimize information leakage between training and validation data. Even with this strategy, the reported metrics represent internal predictive performance within the present experimental space rather than evidence of external generalizability to previously unseen scaffold systems. Independent validation using additional scaffold formulations will therefore be required to determine the broader predictive applicability of the models. The machine learning analysis demonstrated that the multidimensional MMS-QI could be modeled from scaffold-associated descriptors with substantially greater accuracy than the mean baseline. The differences among model classes further suggest that the scaffold–cell relationship contains both relatively simple and potentially nonlinear components. Within the evaluated framework, MMAWE provided the most consistent reconstruction of the integrated cellular response, establishing a computational link between engineered scaffold characteristics and the resulting multidimensional cellular phenotype. These findings provide the basis for subsequent analysis of individual feature contributions and structure–response relationships.

### 3.9. Integrated Cellular Response Profiling and MMS-QI Evaluation

To integrate the multidimensional cellular responses observed across the different experimental conditions, quantitative parameters derived from DAPI, F-actin, and SOX9 analyses were incorporated into the Meniscus-Mimetic Scaffold Quality Index (MMS-QI). The MMS-QI was developed as a study-specific composite metric encompassing three major biological domains: cellularity, meniscus-associated cellular organization, and SOX9-associated phenotype. Individual parameters were normalized prior to integration to account for differences in their measurement scales, and the resulting domain scores were combined to generate a single composite value. Accordingly, a higher MMS-QI represents a relatively greater integrated cellular response within the present experimental dataset rather than an absolute or externally validated measure of scaffold performance.

**Figure 6:**
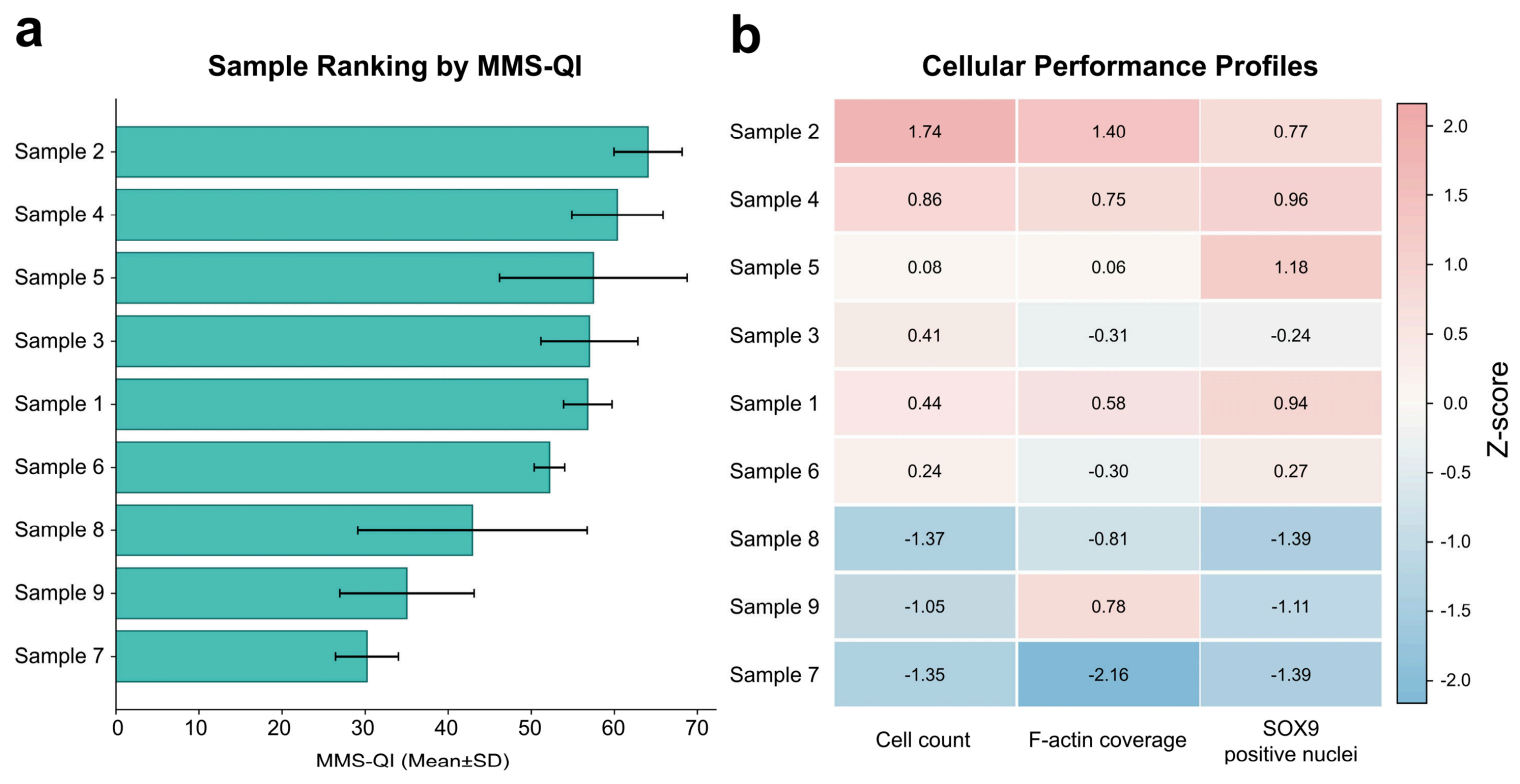
MMS-QI-based sample ordering and cellular performance profiles. (**a**) Group mean Meniscus-Mimetic Scaffold Quality Index (MMS-QI) values with image-level standard deviation (SD). (**b**) Z-score-standardized profiles of representative cellular response features, including cell count, F-actin coverage, and SOX9-positive nuclei, displayed for visualization of relative cellular response patterns across experimental conditions. MMS-QI values were calculated from quantitative fluorescence image features obtained from three independent biological replicates per group (*n* = 3). Four imaging fields were analyzed per replicate. Data are presented as mean ± SD.

The mean MMS-QI values and their image-level variability are presented in Figure 6a and Table 5. Distinct differences in the integrated response were observed among the nine experimental conditions. Sample 2 (PBM-A16) exhibited the highest mean MMS-QI (64.06 ± 4.11), followed by Sample 4 (PBM-R20; 60.38 ± 5.49), Sample 5 (PVDF-R16; 57.48 ± 11.30), Sample 3 (PBM-Rad16; 57.01 ± 5.84), Sample 1 (PBM-R16; 56.82 ± 2.92), and Sample 6 (PVDF-R20; 52.20 ± 1.84). The TCP reference conditions showed lower mean MMS-QI values of 42.93 ± 13.81, 35.04 ± 8.08, and 30.23 ± 3.79 for Samples 8, 9, and 7, respectively. The error bars represent the variability among the analyzed images within each condition and therefore reflect image-level heterogeneity rather than independent biological variability. Overall, the separation in MMS-QI values across the experimental conditions indicates that changes in scaffold environment were accompanied by differences in the combined cellular response captured by the index.

The relative MMS-QI values were not determined by a single cellular parameter. To visualize the underlying response patterns, three representative cellular features—cell count, F-actin coverage, and the proportion of SOX9-positive nuclei—were standardized as Z-scores and displayed as a heatmap (Figure 6b). These features were selected to provide an intuitive representation of cellular abundance, cytoskeletal occupancy, and SOX9-associated phenotype, respectively; they should not be interpreted as equivalent to the complete MMS-QI domains, which incorporated additional quantitative descriptors. For each feature, a positive Z-score indicates a value above the overall dataset mean, whereas a negative Z-score indicates a value below the mean.

The heatmap revealed distinct cellular response profiles among the experimental conditions. Sample 2 displayed positive standardized values across all three representative features, with Z-scores of 1.74 for cell count, 1.40 for F-actin coverage, and 0.77 for SOX9-positive nuclei, demonstrating a relatively balanced profile across the selected cellular endpoints. Other scaffold conditions exhibited more feature-specific patterns. For example, Sample 5 showed cell count and F-actin coverage values close to the dataset mean (Z = 0.08 and 0.06, respectively) but a comparatively high proportion of SOX9-positive nuclei (Z = 1.18). In contrast, Sample 3 exhibited an above-average cell count (Z = 0.41) together with modestly below-average F-actin coverage and SOX9-positive nuclear fraction (Z = −0.31 and −0.24, respectively). These contrasting profiles demonstrate that experimental conditions with comparable integrated MMS-QI values could nevertheless reach those values through different combinations of underlying cellular responses.

The TCP reference conditions displayed a distinct response pattern, particularly in terms of cell count. Samples 7–9 exhibited negative cell-count Z-scores ranging from −1.37 to −1.05, consistent with the comparatively lower nuclear occupancy observed in the DAPI analysis. However, the TCP groups did not exhibit uniformly low values across all features. Notably, Sample 9 showed an above-average F-actin coverage (Z = 0.78) despite below-average cell count and SOX9-positive nuclear fraction. This finding illustrates that a greater F-actin-positive area does not necessarily correspond to greater scaffold-guided cellular organization. As observed in Figure 4, cells on TCP exhibited broad planar spreading, whereas cells on the nanofibrous scaffolds showed architecture-dependent directional organization. F-actin coverage was therefore considered together with alignment- and scaffold–cell correspondence-related parameters within the broader cellular-organization domain rather than being interpreted independently as an indicator of meniscus-mimetic organization.

The composition-matched PBM-R16, PBM-A16, and PBM-Rad16 groups further illustrate the multidimensional nature of the cellular response. Although these scaffolds shared the same PBM formulation and polymer concentration, alteration of the fiber architecture resulted in different MMS-QI values and cellular response profiles. In conjunction with the F-actin observations, these differences indicate that modification of fiber topography altered cellular occupancy and cytoskeletal organization without necessarily producing proportional changes in SOX9-associated responses. Similarly, differences among scaffolds varying in PVDF concentration and composite formulation suggest that material composition and the associated changes in fiber morphology contributed additional sources of variation in the integrated cellular response. Figure 6 demonstrates that scaffold-associated cellular responses were multidimensional and condition dependent. Conditions with relatively high MMS-QI values tended to exhibit favorable responses across multiple cellular parameters, whereas other conditions displayed selective enhancement or reduction of individual features. Thus, the final MMS-QI was determined by the combined contribution of multiple biological characteristics rather than by any single endpoint. Within the scope of the present dataset, the MMS-QI therefore provided a unified quantitative representation of cellularity, nuclear morphology and cytoskeletal organization, and SOX9-associated phenotype, while the accompanying standardized cellular profiles preserved information regarding the distinct biological patterns underlying the composite score. The MMS-QI was subsequently used as the target response variable for machine learning-based modeling of scaffold–cell relationships.

### 3.10. Correlation-Based Analysis of MMS-QI Component Metrics and Model Predictors

**Figure 7:**
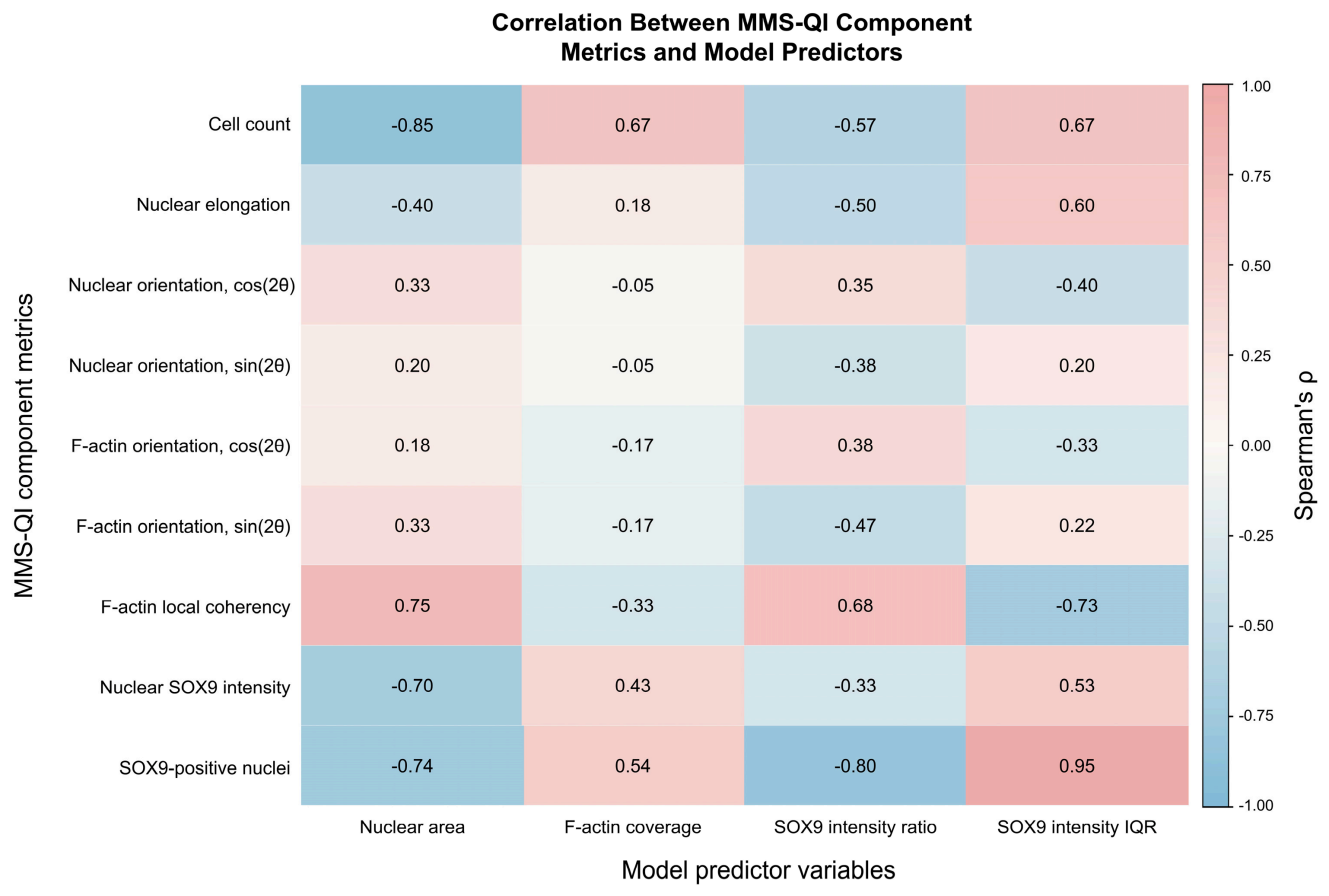
Spearman correlation matrix between MMS-QI source measurements and scaffold predictors used in the MMAWE model.

To further examine the relationships underlying the machine learning-based prediction of the MMS-QI, Spearman rank correlation analysis was performed between the cellular response measurements contributing to the MMS-QI and the scaffold-derived predictors used in the MMAWE model (Figure 7). Unlike the integrated MMS-QI, which summarizes multiple cellular response domains into a composite outcome, this analysis was intended to examine how individual cellular measurements were associated with specific model predictor variables.

The predictor variables used in the MMAWE model were intentionally distinct from the source measurements directly used to construct the MMS-QI. The MMS-QI was derived from cell count, nuclear elongation and orientation, F-actin orientation and local coherency, corrected nuclear SOX9 intensity, and the proportion of SOX9-positive nuclei. In contrast, the predictive model used nuclear area, F-actin coverage, SOX9 intensity ratio, and SOX9 intensity IQR as input variables. Direct MMS-QI source measurements were excluded from the predictor set to reduce circular reuse of variables directly defining the prediction target. Figure 7 therefore examines the associations between the measurements underlying the MMS-QI and the distinct cellular descriptors used for its machine learning-based prediction.

The resulting correlation matrix revealed heterogeneous patterns of positive and negative associations across MMS-QI component–predictor pairs. Spearman correlation coefficients (ρ) ranged from negative to positive values, indicating that increases in a given predictor variable were associated with either decreases or increases in particular cellular responses, depending on the predictor–response combination. Several pairs exhibited relatively large absolute correlation coefficients, whereas others showed weak or near-zero associations. Thus, no single model predictor displayed a uniform relationship with all cellular response measurements.

Several notable associations were observed within the correlation matrix. The proportion of SOX9-positive nuclei showed a strong positive correlation with SOX9 intensity IQR (ρ = 0.95) and a negative correlation with SOX9 intensity ratio (ρ = −0.80). Cell count was negatively correlated with nuclear area (ρ = −0.85), whereas F-actin local coherency showed positive correlations with nuclear area (ρ = 0.75) and SOX9 intensity ratio (ρ = 0.68), but a negative correlation with SOX9 intensity IQR (ρ = −0.73). These relationships indicate that the selected model predictors contained information associated with the biological measurements underlying the MMS-QI, while remaining distinct from the variables directly used in its calculation.

The correlation analysis also provides context for the differences in predictive performance observed among the machine learning models (Table 6). The heterogeneous direction and magnitude of the observed associations suggest that the relationship between the model predictors and the integrated cellular response cannot be readily represented by a single predictor–response association. Instead, MMS-QI prediction likely depends on the combined information provided by multiple predictors, supporting a multivariate relationship in which different cellular descriptors contribute complementary information to the integrated response. This interpretation is consistent with the improved predictive performance achieved by the MMAWE framework, which integrates information across multiple predictors and modeling approaches. Together with the machine learning results, these findings support the use of multidimensional biological descriptors for modeling scaffold-dependent cellular responses within the experimental space investigated in this study.

Nevertheless, the Spearman coefficients shown in Figure 7 represent univariate monotonic associations and should not be interpreted as measures of causal influence or direct estimates of feature importance within the MMAWE model. Correlations among predictor variables, interactions among predictors, and nonlinear predictor–response relationships may all contribute to model predictions but are not captured by pairwise correlation analysis alone. Accordingly, Figure 7 was used as an interpretable complementary analysis of the relationships between MMS-QI component measurements and model predictors rather than as a mechanistic or causal attribution of individual predictor variables.

### 3.11. Predictive Performance and Predictor Importance of the MMAWE Model

**Figure 8:**
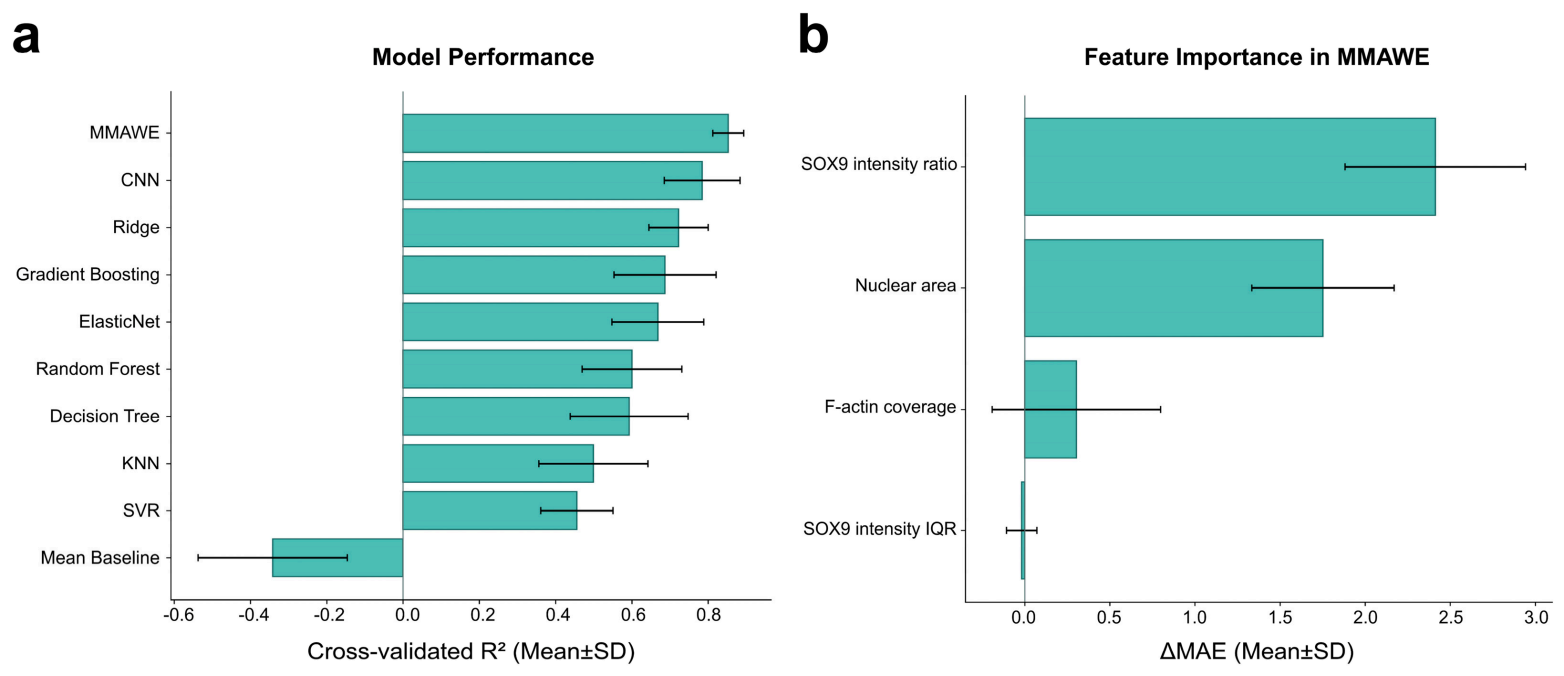
Model performance and predictor importance of the MMAWE model. (**a**) Cross-validated R^2^ comparison between MMAWE and benchmark prediction models. (**b**) Permutation-based predictor importance quantified by the change in mean absolute error (ΔMAE).

Following evaluation of the associations between scaffold characteristics and individual cellular responses, the predictive performance of the machine learning models was further compared using cross-validated coefficients of determination (R^2^) (Figure 8a). The evaluated approaches included conventional linear and nonlinear regression models, tree-based algorithms, a convolutional neural network (CNN), and the proposed MMAWE framework. Model performance was assessed under the same cross-validation framework to enable direct comparison among modeling approaches.

Distinct differences in predictive performance were observed across the evaluated models. Consistent with the quantitative results summarized in Table 6, the mean-prediction baseline showed poor predictive performance, whereas all trained models provided improved prediction of MMS-QI. Among the individual benchmark models, the CNN and Ridge regression exhibited comparatively high cross-validated R^2^ values of 0.784 ± 0.099 and 0.723 ± 0.078, respectively. Gradient Boosting and Elastic Net achieved intermediate performance, whereas Random Forest, Decision Tree, KNN, and SVR showed lower cross-validated R^2^ values within the present dataset.

The MMAWE model achieved the highest mean cross-validated R^2^ among the evaluated approaches (0.853 ± 0.041) (Figure 8a). In addition to its higher mean R^2^, MMAWE exhibited relatively low variability across the cross-validation evaluations. Consistent with this result, MMAWE also showed the lowest prediction errors among the evaluated models, with an MSE of 18.362 and an MAE of 3.386 (Table 6). Together, these results indicate that integration of complementary predictive information within the MMAWE framework improved reconstruction of the integrated MMS-QI within the experimental dataset compared with the individual benchmark models.

To examine the contribution of individual scaffold predictors to MMAWE prediction, permutation-based feature importance was subsequently evaluated (Figure 8b). For each predictor, its values were permuted while the remaining input variables were retained, and the resulting change in prediction error was quantified as the increase in MAE (ΔMAE). A larger positive ΔMAE indicates a greater deterioration in predictive accuracy following disruption of that predictor, suggesting that the trained model relied more strongly on the information contained in that variable.

The permutation analysis revealed an unequal contribution of the scaffold predictors to MMS-QI prediction. Disruption of the highest-ranked predictors produced substantially greater increases in MAE than disruption of the lower-ranked variables, indicating that the predictive information available to MMAWE was concentrated more strongly in particular scaffold descriptors. Conversely, predictors producing little or no increase in MAE contributed comparatively little unique predictive information under the fitted model and validation framework.

Permutation importance reflects model dependence on predictor information rather than direct biological causality. Scaffold descriptors may be correlated with one another, and permutation of one variable can therefore alter information that is partially shared with other predictors. Furthermore, nonlinear interactions among scaffold characteristics may contribute to MMAWE predictions even when an individual variable exhibits only modest univariate correlation with the MMS-QI source measurements. Accordingly, the predictor importance results were interpreted together with the Spearman correlation analysis in Figure 7 rather than as independent evidence of a causal hierarchy among scaffold properties.

The complementary information provided by Figure 7 and Figure 8 therefore offers two different perspectives on scaffold–cell relationships. Whereas the Spearman analysis describes pairwise monotonic associations between individual scaffold characteristics and cellular response measurements, permutation importance evaluates how strongly the trained MMAWE model depends on each predictor for accurate MMS-QI prediction. Differences between these two analyses are therefore expected and may reflect multivariable interactions, redundancy among predictors, or nonlinear relationships that cannot be captured by pairwise correlations alone.

The cross-validated performance and permutation analyses demonstrate that MMS-QI prediction was improved by integrating information from multiple scaffold characteristics and modeling strategies. Within the present experimental space, MMAWE provided the highest predictive performance among the evaluated models, while permutation analysis identified differential contributions of the scaffold-derived predictors to model prediction. These results support a multivariate structure–response framework in which integrated cellular behavior is associated with the combined structural characteristics of the engineered nanofibrous scaffolds rather than with a single scaffold parameter.

### 3.12. Sample-Level Prediction Errors and Observed–Predicted Agreement

**Figure 9:**
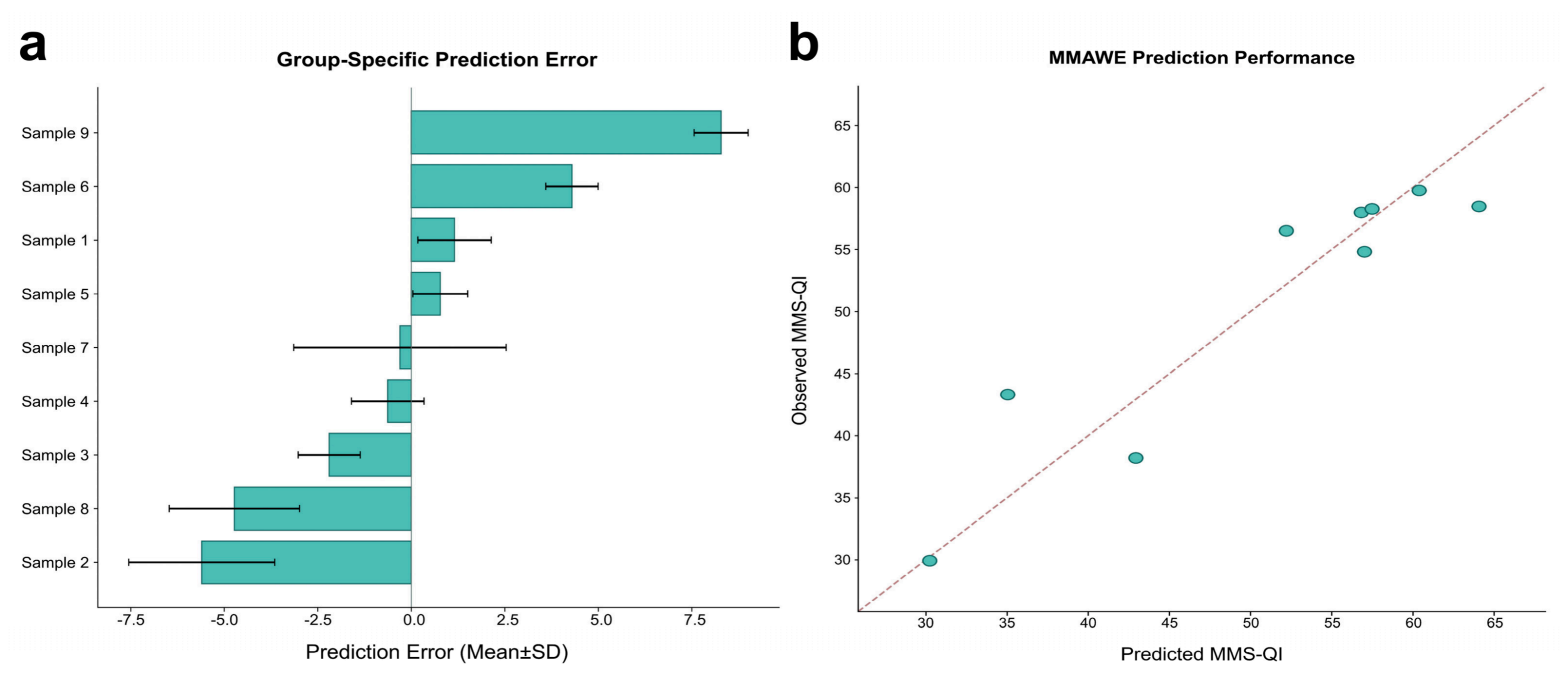
Predictive performance of the MMAWE model. (**a**) Prediction errors calculated as predicted minus observed MMS-QI. (**b**) Agreement between observed and predicted MMS-QI values. The dashed line represents the identity line (y = x), indicating perfect agreement between predicted and observed values. Data points closer to this line indicate higher prediction accuracy, whereas larger deviations reflect greater prediction errors.

To further evaluate the predictive behavior of the MMAWE model beyond the aggregate performance metrics, sample-level prediction errors and the agreement between observed and predicted MMS-QI values were examined (Figure 9). This analysis was performed to determine whether the overall predictive performance of MMAWE was consistently distributed across the experimental conditions or whether systematic over- or underprediction occurred for particular samples.

Figure 9a shows the prediction error for each experimental condition, calculated as the difference between the predicted and observed MMS-QI values (predicted–observed). Accordingly, positive values indicate overprediction of the experimentally derived MMS-QI, whereas negative values indicate underprediction. The magnitude of the error varied among experimental conditions, demonstrating that the predictive accuracy of MMAWE was not uniform across all samples. Several conditions showed relatively small deviations from zero, whereas others exhibited larger positive or negative errors. The error bars further illustrate variability in prediction error across the corresponding cross-validation evaluations.

Prediction errors occurred in both directions rather than exclusively as overprediction or underprediction. This pattern suggests that the model did not simply introduce a uniform directional offset across the MMS-QI range. Nevertheless, the presence of larger sample-specific deviations indicates that certain scaffold conditions were more difficult to predict from the available scaffold descriptors. Such deviations may reflect condition-specific cellular responses that were not fully represented by the predictor set, interactions among scaffold characteristics, or the limited experimental diversity available for model training.

The relationship between observed and predicted MMS-QI values is shown in Figure 9b. The diagonal identity line represents perfect agreement between model prediction and experimentally derived MMS-QI. Most observations followed the overall direction of the identity line, indicating that MMAWE captured the relative variation in MMS-QI across experimental conditions. This observation is consistent with the cross-validated performance reported in Table 6 and Figure 8.

However, deviations from the identity line remained evident for individual samples, particularly within portions of the MMS-QI range where predicted values tended to be compressed relative to the experimentally observed values. This behavior indicates that, although the model captured the overall structure of the response, it did not reproduce every condition with equal accuracy. Such sample-level discrepancies are important given the relatively limited number of experimental conditions and reinforce the interpretation of MMAWE as an exploratory predictive framework within the present experimental space, rather than as an externally validated model for arbitrary scaffold systems.

The sample-level error analysis and observed–predicted comparison complement the aggregate R^2^, MSE, and MAE metrics by providing a more direct assessment of model behavior across individual experimental conditions. The results indicate that MMAWE captured the overall variation in MMS-QI while retaining condition-dependent prediction errors, thereby defining both the predictive capability and the current limitations of the model. Expansion of the training space to include additional scaffold architectures and compositions will be required to determine whether these sample-specific errors can be reduced and whether the observed predictive relationships generalize to previously unseen scaffold systems.

### 3.13. SHAP-Based Interpretation of MMAWE Predictions

**Figure 10:**
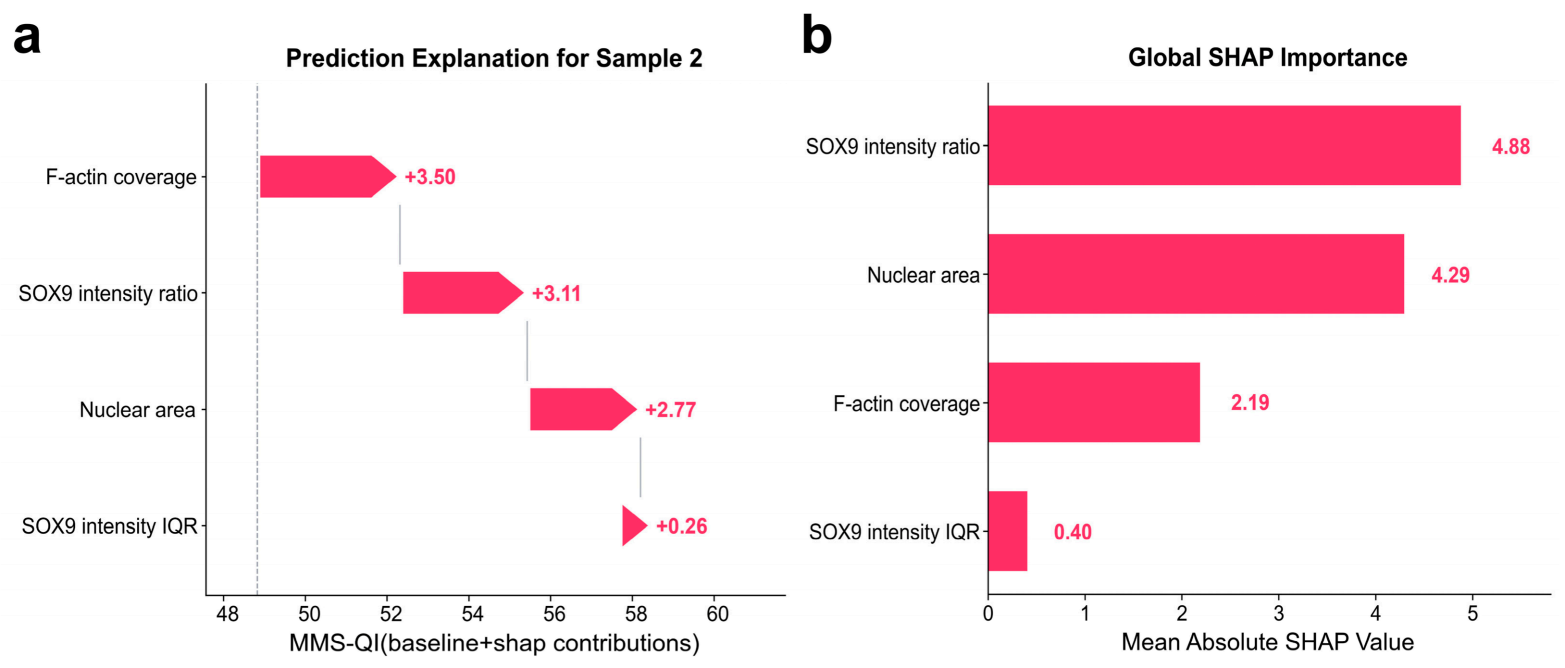
SHAP-based feature contributions of the MMAWE model. (**a**) SHAP waterfall plot showing feature-level contributions to the predicted MMS-QI for Sample 2 (PBM-A16). Positive SHAP values indicate contributions that increase the predicted MMS-QI relative to the reference prediction. (**b**) Global feature importance across the analyzed samples, quantified as the mean absolute SHAP value (mean |SHAP|) for each input feature.

Although permutation importance identified the variables on which MMAWE relied for accurate prediction, it did not indicate the direction or magnitude of each feature’s contribution to an individual MMS-QI prediction. To provide a more detailed interpretation of model behavior, SHapley Additive exPlanations (SHAP) analysis was therefore performed to quantify feature-level contributions to MMAWE predictions (Figure 10). SHAP values describe the extent to which each input feature shifts a model prediction relative to a reference prediction, with positive and negative values indicating contributions toward higher and lower predicted MMS-QI values, respectively.

Given the limited number of input variables, feature contributions were calculated by evaluating the possible feature combinations used to determine the marginal contribution of each variable. To minimize information leakage during interpretation, each sample being explained was treated as validation data, while the reference value used for SHAP estimation was derived exclusively from the corresponding training groups. Thus, the resulting SHAP values reflect model behavior under the group-separated validation framework rather than explanations derived from a model fitted using the sample being interpreted.

A local SHAP explanation for Sample 2 (PBM-A16) is presented in Figure 10a. Sample 2 was selected because it exhibited the highest experimentally determined MMS-QI and was also assigned a comparatively high MMS-QI by MMAWE, providing a representative case for examining how the model reconstructed a high-response condition. Starting from the reference prediction, all four analyzed features contributed positively to the predicted MMS-QI for this sample. The largest positive contribution was associated with F-actin coverage (+3.50 MMS-QI units), followed by the other feature contributions of +3.11, +2.77, and +0.26, respectively. Collectively, these positive contributions shifted the prediction upward from the reference level toward the Sample 2-specific predicted MMS-QI.

The local explanation is particularly informative because it demonstrates that the high predicted MMS-QI for PBM-A16 was not attributable to a single model input. Rather, multiple features contributed in the same direction, although with substantially different magnitudes. The comparatively large contribution associated with F-actin coverage indicates that this feature provided substantial information for the Sample 2 prediction within the fitted MMAWE model. Importantly, however, a positive SHAP value should be interpreted as a model-specific contribution to the prediction, rather than evidence that increasing the corresponding biological variable would causally increase MMS-QI.

To complement this sample-specific interpretation, global SHAP importance was calculated across the analyzed samples using the mean absolute SHAP value for each input feature (Figure 10b). Unlike signed local SHAP values, mean absolute SHAP values quantify the average magnitude by which each feature altered model predictions regardless of the direction of its effect. The SOX9 nuclear-to-background intensity ratio showed the largest global contribution (mean |SHAP| = 4.88), followed by nuclear area (4.29), F-actin coverage (2.19), and the SOX9 signal-distribution width (0.40). Thus, the relative contribution observed for a particular sample did not necessarily correspond to the overall importance of the same variable across the complete dataset.

This distinction between local and global explanations is exemplified by F-actin coverage. Although F-actin coverage produced the largest positive contribution to the Sample 2 prediction, it ranked below the SOX9 nuclear-to-background ratio and nuclear area when importance was averaged across all evaluated samples. Conversely, the SOX9 nuclear-to-background ratio exhibited the largest mean absolute SHAP value globally, indicating that variation in this feature produced comparatively large changes in MMAWE predictions across the dataset. These findings emphasize that sample-specific predictive mechanisms can differ from the average behavior of the model across experimental conditions.

The SHAP results also complement the permutation-based analysis presented in Figure 8b. Permutation importance evaluates the deterioration in overall predictive performance when the information contained in a predictor is disrupted, whereas SHAP analysis decomposes individual predictions into feature-level contributions and provides both their magnitude and direction. Agreement between these approaches can strengthen evidence that a feature contains substantial predictive information, whereas differences are not necessarily contradictory because the two methods quantify different aspects of model behavior.

Nevertheless, the SHAP results should be interpreted within the limitations of the present dataset. Feature contributions describe how the fitted MMAWE model generated its predictions and do not establish causal relationships between individual cellular characteristics and MMS-QI. Moreover, correlated input variables can influence the allocation of SHAP contributions among features. Accordingly, the SHAP analysis was used primarily to enhance model transparency and to identify the variables contributing to individual and global predictions within the current experimental space.

The local and global SHAP analyses demonstrated that MMAWE predictions were generated through the combined contributions of multiple cellular features rather than through reliance on a single variable. The Sample 2 analysis provided an interpretable example of how several features collectively shifted the prediction toward a high MMS-QI, whereas the global analysis identified the features that most strongly influenced predictions across the dataset. Together with the permutation importance and correlation analyses, these results provide complementary levels of interpretation of the multivariate relationships underlying MMAWE-based MMS-QI prediction.

### 3.14. Group-Specific SHAP Contributions and Directionality of MMAWE Predictions

**Figure 11:**
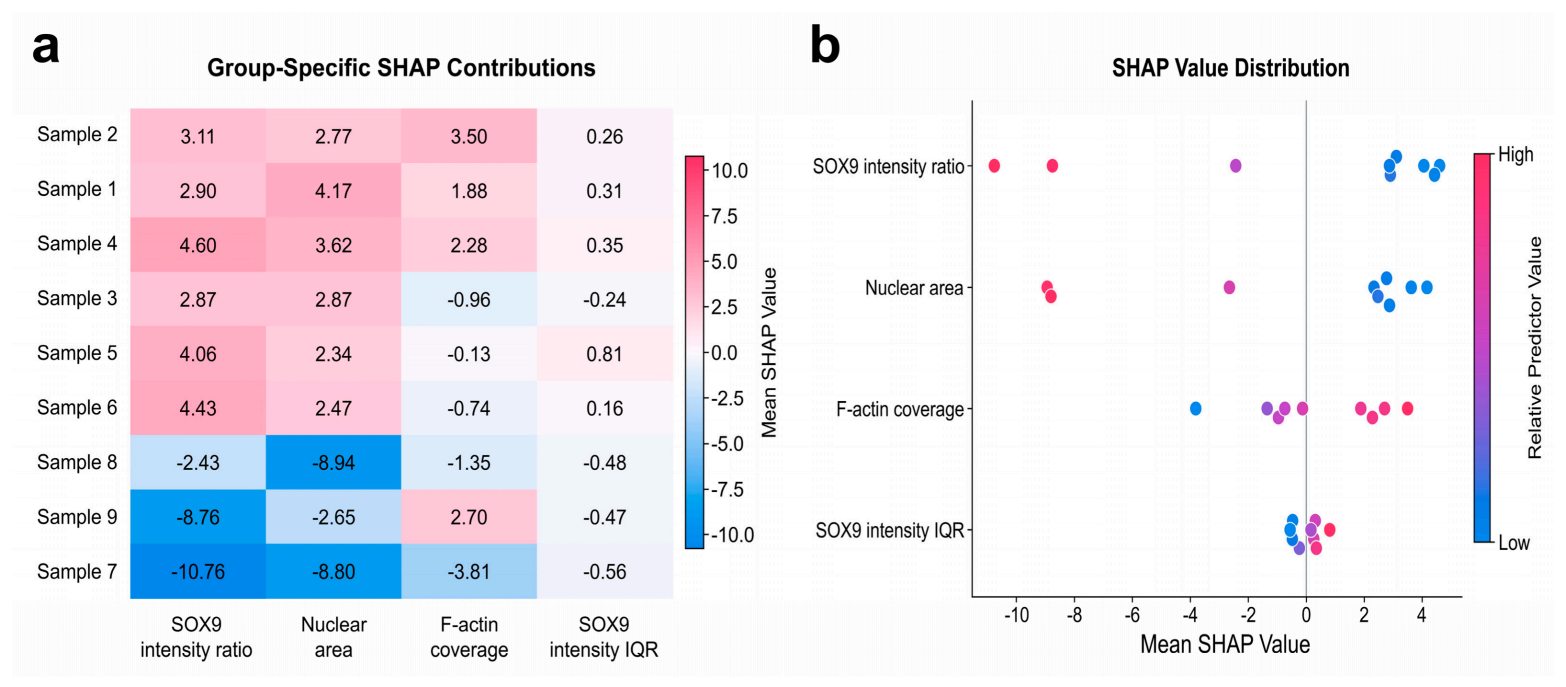
Group-specific SHAP interpretation of MMAWE predictions. (**a**) Heatmap of signed SHAP values showing the magnitude and direction of individual predictor contributions across experimental groups. (**b**) Distribution of group-specific SHAP values for each predictor, with point color indicating the relative predictor value. Positive and negative SHAP values indicate contributions toward higher and lower predicted MMS-QI values, respectively.

To further investigate whether the contribution of individual predictors was consistent across experimental conditions, SHAP analysis was extended to all nine groups (Figure 11). Whereas the global mean absolute SHAP analysis in Figure 10b quantified the overall magnitude of predictor importance irrespective of direction, the group-specific analysis retained the sign of each SHAP value, thereby allowing both the magnitude and direction of predictor contributions to be compared across experimental conditions.

The group-level SHAP heatmap revealed substantial heterogeneity in predictor contributions among the nine experimental conditions (Figure 11a). Positive SHAP values indicate that a predictor shifted the MMAWE prediction toward a higher MMS-QI relative to the corresponding reference prediction, whereas negative values indicate a contribution toward a lower predicted MMS-QI. Importantly, the same predictor did not exert a uniform directional contribution across all groups. Instead, both the magnitude and sign of its contribution varied depending on the experimental condition, demonstrating that MMAWE predictions were generated from group-specific combinations of predictor information.

This pattern was particularly evident for the SOX9 nuclear-to-background intensity ratio and nuclear area, which also showed the largest global mean absolute SHAP values in Figure 10b. For the scaffold groups with relatively high MMS-QI values, including Samples 1, 2, 4, 5, and 6, these predictors generally contributed positively to the predicted MMS-QI. In contrast, several TCP reference conditions exhibited substantial negative contributions from the same predictors. For example, the SOX9 intensity ratio showed a strongly negative contribution in Sample 7, while nuclear area contributed strongly negatively in Samples 7 and 8. These contrasting contributions indicate that predictors identified as globally important did not simply shift all predictions in the same direction; rather, their contribution depended on the feature profile of each experimental condition.

F-actin coverage exhibited a similarly condition-dependent pattern. It contributed positively to the Sample 2 prediction, consistent with the local SHAP analysis shown in Figure 10a, and also showed positive contributions in several other conditions. In contrast, negative contributions were observed for Samples 3, 5, 6, 7 and 8. The SOX9 intensity interquartile range (IQR) generally exhibited smaller SHAP magnitudes, consistent with its relatively low global importance in Figure 10b, although the direction of its contribution likewise varied among groups. Together, these results demonstrate that global predictor importance alone does not fully describe how MMAWE generated individual predictions.

The distribution of group-specific SHAP values is further illustrated in Figure 11b. Each point represents the SHAP contribution of a predictor for an individual experimental group, with its horizontal position indicating the direction and magnitude of the contribution to the predicted MMS-QI. Points located to the right of zero contributed toward higher predicted MMS-QI values, whereas those to the left contributed toward lower values. The color of each point represents the relative magnitude of the corresponding predictor value, enabling the relationship between the underlying feature value and its contribution to model prediction to be examined simultaneously.

The resulting distributions further illustrate that the relationship between predictor magnitude and model contribution was not identical across all features. For some predictors, relatively high and low feature values were associated with clearly different SHAP directions, whereas other predictors showed values clustered around zero or more heterogeneous contribution patterns. This behavior indicates that the predictive contribution of a variable cannot necessarily be inferred from its raw magnitude alone and may depend on the multivariate context represented within the MMAWE model.

The complementary SHAP visualizations in Figure 10 and Figure 11 therefore provide three levels of model interpretation. The local waterfall analysis illustrates how individual predictors collectively generated a specific prediction; mean absolute SHAP values summarize the overall magnitude of predictor influence across the dataset; and the group-specific SHAP analysis reveals how the direction and magnitude of those contributions varied among experimental conditions. This distinction is important because a predictor with high global importance may contribute positively to some predictions and negatively to others.

These findings are also consistent with the multidimensional cellular response patterns observed in Figure 6. Experimental conditions differed not only in their integrated MMS-QI values but also in the combinations of cellular features underlying those values. The SHAP analysis extends this observation to the predictive model by demonstrating that MMAWE did not rely on a single fixed predictor hierarchy or directional rule across all experimental groups. Rather, the predicted MMS-QI emerged from condition-specific combinations of feature contributions.

As with the preceding model-interpretation analyses, SHAP values describe the behavior of the fitted predictive model and should not be interpreted as evidence of biological causality. Nevertheless, the group-specific analysis improves model transparency by demonstrating how individual predictors contributed differently across the experimental conditions. Collectively, Figure 10 and Figure 11 indicate that MMAWE captured a heterogeneous and multivariate relationship between the measured cellular features and integrated MMS-QI within the present experimental dataset.

## 4. Conclusions

In this study, we established an integrated experimental–computational framework to quantitatively evaluate topography-dependent cellular responses to meniscus-mimetic PVDF-based nanofibrous scaffolds. Controlled electrospinning enabled the fabrication of random, aligned, and radial fibrous architectures, while the incorporation of BTO and MWCNTs and variation in PVDF concentration introduced additional compositional and morphological diversity. Quantitative analyses of DAPI, F-actin, and SOX9 fluorescence images demonstrated scaffold-dependent differences in cellular occupancy, nuclear morphology, cytoskeletal organization, scaffold–cell directional correspondence, and SOX9-associated phenotype.

Among the evaluated scaffold groups, the aligned PVDF/BTO/MWCNT scaffold fabricated using 16 wt% PVDF (PBM-A16) exhibited the highest mean Meniscus-Mimetic Scaffold Quality Index (MMS-QI) of 64.06 ± 4.11, compared with 56.82 ± 2.92 for the composition-matched random scaffold (PBM-R16) and 57.01 ± 5.84 for the radial scaffold (PBM-Rad16). These values represented approximately 12.7% and 12.4% higher mean MMS-QI scores, respectively, demonstrating that fiber architecture was associated with distinct multidimensional cellular responses even when scaffold composition and polymer concentration were maintained constant. Notably, the radial scaffold exhibited the highest meniscus-organization domain score (0.75 ± 0.02), whereas the aligned scaffold showed a comparably high organization score (0.73 ± 0.03) together with a higher cellularity score (0.87 ± 0.10). These findings indicate that different fiber architectures produced distinct combinations of cellular responses rather than uniformly enhancing all biological parameters.

Machine learning analysis further demonstrated the feasibility of modeling the integrated cellular responses using quantitative image-derived descriptors. The proposed MMAWE model achieved the highest cross-validated predictive performance, with an R^2^ of 0.853 ± 0.041, an MSE of 18.362, and an MAE of 3.386. Compared with the strongest individual benchmark model (CNN; R^2^ = 0.784 ± 0.099), MMAWE reduced the MSE by approximately 31.7% and the MAE by approximately 13.3%. Permutation-based feature importance and SHAP analyses further revealed that predictions were influenced by multiple cellular descriptors, with the SOX9 nuclear-to-background intensity ratio and nuclear area exhibiting the highest global mean absolute SHAP values of 4.88 and 4.29, respectively. These findings highlight the potential of integrating multidimensional cellular measurements with interpretable machine learning to characterize scaffold-dependent biological responses.

Collectively, these results demonstrate that scaffold architecture and material composition are associated with distinct cellular response profiles that cannot be adequately represented by a single biological endpoint. The integration of quantitative fluorescence imaging, MMS-QI evaluation, and interpretable machine learning provides a systematic approach for assessing the relationships between engineered fibrous microenvironments and cellular behavior. Nevertheless, given the limited number of scaffold formulations and the study-specific nature of the MMS-QI, the predictive framework should be considered exploratory rather than broadly generalizable. Future studies incorporating additional scaffold formulations, independent biological validation, functional and extracellular matrix-associated markers, and electromechanical characterization will be necessary to establish broader predictive applicability. The present findings provide a quantitative foundation for the rational evaluation and optimization of meniscus-mimetic nanofibrous scaffolds.

### Statistical Analysis

Data are presented as mean ± standard error of the mean (SEM). For comparisons between two groups, statistical significance was evaluated using an unpaired two-tailed Student’s *t*-test. For multiple group comparisons (≥3 groups), one-way ANOVA followed by Tukey’s post hoc test was applied. A *p*-value < 0.05 was considered statistically significant. Significance levels were denoted as * *p* < 0.05, ** *p* < 0.01, and *** *p* < 0.001 versus the control group, unless otherwise specified.

## Figures and Tables

**Table 1:** Experimental scaffold groups and TCP reference conditions used for quantitative cellular imaging.

Sample 1	PBM-R16
Sample 2	PBM-A16
Sample 3	PBM-Rad16
Sample 4	PBM-R20
Sample 5	PVDF-R16
Sample 6	PVDF-R20
Sample 7	TCP 5000
Sample 8	TCP 10,000
Sample 9	TCP 20,000

**Table 2:** Summary statistics of DAPI-derived nuclear features quantified from hBMSCs cultured on nanofibrous scaffolds and TCP.

Feature	Mean ± SD	Min	Max
Cell count	137.04 ± 50.10	58	231
Median nuclear elongation	1.496 ± 0.073	1.388	1.680
Dominant nuclear orientation (°)	13.60 ± 54.24	3.37	167.31
Median nuclear area (µm^2^)	0.713 ± 0.108	0.554	1.053
Nuclear alignment index (0–1)	0.266 ± 0.168	0.037	0.607

**Table 3:** Summary statistics of F-actin-derived cytoskeletal features quantified from hBMSCs cultured on nanofibrous scaffolds and TCP.

Feature	Mean ± SD	Min	Max
Dominant F-actin orientation (°)	65.77 ± 50.36	26.47	164.98
F-actin local coherency (0–1)	0.513 ± 0.070	0.394	0.695
F-actin alignment index (0–1)	0.394 ± 0.282	0.043	0.934
F-actin coverage (%)	26.61 ± 5.78	16.85	38.75

**Table 4:** Summary statistics of nuclear SOX9 expression features quantified from hBMSCs cultured under the experimental conditions.

Feature	Mean ± SD	Min	Max
SOX9-positive nuclei (%)	29.30 ± 14.24	3.90	58.73
Median background-corrected nuclear SOX9 intensity (AU)	5.871 ± 4.911	−0.473	19.997
IQR of background-corrected nuclear SOX9 intensity (AU)	21.220 ± 11.695	7.488	51.160
Median nuclear-to-background SOX9 intensity ratio	1.055 ± 0.039	0.995	1.124
Median robust SOX9 signal-to-noise ratio	0.163 ± 0.106	−0.018	0.354
SOX9-positive nuclei (%)	29.30 ± 14.24	3.90	58.73

**Table 5:** Sample ranking and domain scores of the meniscus-mimetic quality index (MMS-QI).

Rank	Sample	MMS-QI (Mean ± SD)	Cellularity (Mean ± SD)	Meniscus Organization (Mean ± SD)	SOX9 Expression (Mean ± SD)	n
1	Sample 2	64.06 ± 4.11	0.87 ± 0.10	0.73 ± 0.03	0.41 ± 0.03	4
2	Sample 4	60.38 ± 5.49	0.76 ± 0.21	0.68 ± 0.08	0.44 ± 0.23	4
3	Sample 5	57.48 ± 11.30	0.65 ± 0.12	0.64 ± 0.18	0.47 ± 0.22	4
4	Sample 3	57.01 ± 5.84	0.74 ± 0.18	0.75 ± 0.02	0.30 ± 0.13	4
5	Sample 1	56.82 ± 2.92	0.73 ± 0.15	0.62 ± 0.03	0.41 ± 0.13	4
6	Sample 6	52.20 ± 1.84	0.72 ± 0.12	0.56 ± 0.06	0.34 ± 0.10	4
7	Sample 8	42.93 ± 13.81	0.20 ± 0.07	0.70 ± 0.07	0.40 ± 0.40	4
8	Sample 9	35.04 ± 8.08	0.36 ± 0.03	0.52 ± 0.25	0.22 ± 0.03	4
9	Sample 7	30.23 ± 3.79	0.21 ± 0.10	0.55 ± 0.03	0.19 ± 0.01	4

**Table 6:** Performance comparison of multiple machine-learning models and a baseline model for predicting the Meniscus-Mimetic Scaffold Quality Index (MMS-QI).

Model	R^2^ (Mean ± SD)	MSE	MAE
Ridge Regression	0.723 ± 0.078	34.590	4.648
Elastic Net	0.668 ± 0.120	41.381	5.035
Random Forest	0.600 ± 0.130	49.829	5.407
Gradient Boosting	0.687 ± 0.134	39.020	5.064
Decision Tree	0.593 ± 0.154	50.764	6.140
K-Nearest Neighbors (KNN)	0.499 ± 0.143	62.467	5.956
Support Vector Regression (SVR)	0.456 ± 0.095	67.839	6.311
Convolutional Neural Network (CNN)	0.784 ± 0.099	26.894	3.907
Mean Baseline	−0.342 ± 0.196	167.290	11.122
MMAWE	0.853 ± 0.041	18.362	3.386

## Data Availability

The original contributions presented in the study are included in the article, further inquiries can be directed to the corresponding authors.
